# Metabolic division of labor drives estuarine-coastal N_2_O emissions

**DOI:** 10.1093/ismejo/wrag216

**Published:** 2026-08-26

**Authors:** Enquan Zhang, Shengjie Li, Ehui Tan, Qiuyun Jiang, Yunxuan Li, Ziqi Wu, Jiaxin Chen, Xianhui Wan, Xianbiao Lin, Nengwang Chen, Yu Cong, Nianzhi Jiao, Xiyang Dong, Qiang Zheng

**Affiliations:** Department of Marine Biology and Technology, College of Ocean and Earth Sciences and State Key Laboratory of Marine Environmental Science, Xiamen University, Xiamen 361005, China; Fujian Key Laboratory of Marine Carbon Sequestration, Xiamen University, Xiamen 361005, China; State Key Laboratory of Submarine Geoscience, and School of Oceanography, Shanghai Jiao Tong University, Shanghai 200030, China; State Key Laboratory of Marine Resource Utilization in South China Sea, Hainan University, Haikou 570228, China; Key Laboratory of Marine Genetic Resources, Third Institute of Oceanography, Ministry of Natural Resources, Xiamen 361005, China; Department of Marine Biology and Technology, College of Ocean and Earth Sciences and State Key Laboratory of Marine Environmental Science, Xiamen University, Xiamen 361005, China; Fujian Key Laboratory of Marine Carbon Sequestration, Xiamen University, Xiamen 361005, China; Department of Marine Biology and Technology, College of Ocean and Earth Sciences and State Key Laboratory of Marine Environmental Science, Xiamen University, Xiamen 361005, China; Fujian Key Laboratory of Marine Carbon Sequestration, Xiamen University, Xiamen 361005, China; Department of Marine Biology and Technology, College of Ocean and Earth Sciences and State Key Laboratory of Marine Environmental Science, Xiamen University, Xiamen 361005, China; Fujian Key Laboratory of Marine Carbon Sequestration, Xiamen University, Xiamen 361005, China; Department of Marine Biology and Technology, College of Ocean and Earth Sciences and State Key Laboratory of Marine Environmental Science, Xiamen University, Xiamen 361005, China; Frontiers Science Center for Deep Ocean Multispheres and Earth System and Key Laboratory of Marine Chemistry Theory and Technology, Ministry of Education, Ocean University of China, Qingdao 266100, China; Department of Marine Biology and Technology, College of Ocean and Earth Sciences and State Key Laboratory of Marine Environmental Science, Xiamen University, Xiamen 361005, China; Department of Marine Biology and Technology, College of Ocean and Earth Sciences and State Key Laboratory of Marine Environmental Science, Xiamen University, Xiamen 361005, China; Fujian Key Laboratory of Marine Carbon Sequestration, Xiamen University, Xiamen 361005, China; Department of Marine Biology and Technology, College of Ocean and Earth Sciences and State Key Laboratory of Marine Environmental Science, Xiamen University, Xiamen 361005, China; Fujian Key Laboratory of Marine Carbon Sequestration, Xiamen University, Xiamen 361005, China; Key Laboratory of Marine Genetic Resources, Third Institute of Oceanography, Ministry of Natural Resources, Xiamen 361005, China; Department of Marine Biology and Technology, College of Ocean and Earth Sciences and State Key Laboratory of Marine Environmental Science, Xiamen University, Xiamen 361005, China; Fujian Key Laboratory of Marine Carbon Sequestration, Xiamen University, Xiamen 361005, China

**Keywords:** nitrous oxide, salinity gradient, incomplete denitrification, microbial interactions, NO/N_2_O exchange gap, metagenomics, metatranscriptomics, genome-scale metabolic models

## Abstract

Estuarine and coastal systems are global hotspots of marine nitrous oxide (N_2_O) emissions, where microbial nitrification and denitrification are the primary processes regulating N_2_O dynamics. However, how interactions among different N_2_O-associated microorganisms influence ecosystem-scale N_2_O emissions remains poorly understood. This study combined *in situ* N_2_O concentrations, ^15^N-based potential rates, metagenomics, metatranscriptomics, and genome-scale metabolic model analysis to explore N_2_O production and reduction processes in estuarine and coastal systems. Potential N_2_O production and reduction rates, together with *in situ* concentrations, the relative abundance, and the transcriptional activity of associated genes, were significantly higher at low salinity and declined toward coastal regions. Based on the gene content of 974 recovered N_2_O-associated genomes, microorganisms were classified into three functional groups: net N_2_O producers, net N_2_O consumers, and self-sustaining N_2_O players. The abundance, composition, and activity of these functional groups shifted along estuarine-coastal gradients. A larger NO/N_2_O exchange gap, reflecting the imbalance between model-inferred NO and N_2_O handoff potentials, was found at low salinity and was associated with elevated bottom-water N_2_O concentrations. Together, community-level division of labor and the associated exchange gap provide a conceptual framework for linking N_2_O-related functional groups to N_2_O accumulation in estuarine-coastal ecosystems.

## Introduction

Nitrous oxide (N_2_O) is a potent greenhouse gas with a 100-year global warming potential 265–298 times that of CO_2_ and contributes ~6%–7% of anthropogenic global warming [1–3]. The global ocean represents the largest natural aquatic source of atmospheric N_2_O, contributing around 3.5 (2.5–4.7) Tg N-N_2_O y^−1^ [4, 5]. Estuarine-coastal zones, the dynamic interface between terrestrial and marine environments, are characterized by strong riverine inputs, steep physicochemical gradients, high organic matter supply, and intense nitrogen transformation [6]. In these systems, watershed-derived and sediment-regenerated reactive nitrogen, particularly ammonia (NH_4_^+^) and nitrate (NO_3_^−^), provides substrates for microbial N_2_O production [7]. Anthropogenic nutrient loading can further increase this substrate supply and modify organic matter and oxygen conditions, making estuarine-coastal sediments important hotspots of N_2_O emissions [8, 9].

In ecosystems, N_2_O dynamics are regulated primarily by two microbially mediated processes, nitrification and denitrification [10]. Nitrification is performed by ammonia-oxidizing bacteria (AOB), complete ammonia oxidizers (comammox *Nitrospira*), and ammonia-oxidizing archaea (AOA). AOB and comammox oxidize NH_4_^+^ to hydroxylamine via ammonia monooxygenase (AMO, encoded by *amoA*), and hydroxylamine is subsequently converted to nitrite by hydroxylamine oxidoreductase; non-enzymatic reactions of hydroxylamine can yield N_2_O [11]. Although AOA do not have hydroxylamine oxidoreductase, they still produce small amounts of N_2_O through mechanisms that remain unresolved [12].

Denitrification stepwise reduces NO_3_^−^ to nitrite (NO_2_^−^), nitric oxide (NO), N_2_O, and ultimately dinitrogen (N_2_). N_2_O is produced during NO reduction to N_2_O by nitric oxide reductase (NOR, encoded by *nor*) [13]. Three NORs have been biochemically characterized: the cytochrome-c-dependent cNOR, the quinol-dependent qNOR, and the *Bacillales*-associated Cu_A_-containing NOR (often termed bNOR) [14]. Subsequent work identified four new clades of NORs (eNOR, sNOR, gNOR, and nNOR), with NO reduction activity experimentally confirmed for eNOR [13]. N_2_O is further reduced to N_2_ by nitrous oxide reductase (N_2_OR, encoded by *nosZ*), the only known enzymatic sink for N_2_O [15]. The *nosZ* gene is classified into two major clades: clade I has mainly been associated with *Pseudomonadota*, whereas clade II spans a broader taxonomic and environmental range [15]. The discovery of clade III *nosZ* expands the known diversity of N_2_O-reducing microorganisms and has been detected across multiple phyla and geographical regions [16], but its contribution to N_2_O reduction in estuarine-coastal sediments remains unresolved. Because the genetic capacities for N_2_O production and reduction are often distributed among different microorganisms, ecosystem-level N_2_O dynamics may depend not only on individual genes or taxa but also on the organization of functionally complementary populations.

Recent omics-based studies have shown that complete denitrification in nature rarely occurs within a single organism. Instead, denitrification is often distributed across microorganisms that encode only subsets of the pathway, making NO_x_ transformation a community-level trait [17, 18]. Such pathway fragmentation creates dependence on metabolic handoffs, because NO and N_2_O produced by one organism may be further transformed by other community members with different reduction capacities [17, 19, 20]. Importantly, N_2_O-producing and N_2_O-reducing populations may respond in parallel to the same environmental conditions, such that their individual abundance or activity alone may not explain whether N_2_O ultimately accumulates. A community-level measure of the relative potentials for upstream NO handoff and downstream N_2_O handoff may therefore provide additional insight into the organization of microbial processes associated with N_2_O production and reduction. However, it remains unclear how this functional organization varies across estuarine-coastal gradients and whether the imbalance between these handoff potentials is associated with field N_2_O accumulation. We therefore tested two linked hypotheses: first, environmental gradients would reorganize the relative abundance and composition of N_2_O-associated functional groups in estuarine-coastal sediments; and second, community-level metabolic division of labor would alter the relative potentials for NO and N_2_O handoff, with this imbalance being associated with N_2_O dynamics.

To address the two hypotheses, we investigated field N_2_O concentrations, isotope-based potential rates, metagenomics, and metatranscriptomics across estuarine-coastal environmental gradients along the northwestern Pacific margin (24.4–31.7° N, 117.8–125.9° E). We quantified surface- and bottom-water N_2_O concentrations across 95 sampling sites, measured potential N_2_O production and reduction rates along a salinity gradient in the Jiulong River Estuary (JRE), and characterized N_2_O-related genes and microorganisms using 165 sediment metagenomes and 35 metatranscriptomes. Genome-scale metabolic modeling (GEM) analysis was applied to understand metabolic interactions among N_2_O-associated microorganisms. By integrating these datasets, we evaluated how environmental gradients reorganize N_2_O-related functional groups and the inferred metabolic division of labor among these groups across estuarine-coastal ecosystems.

## Materials and methods

### Study area and sample collection

**Figure 1:**
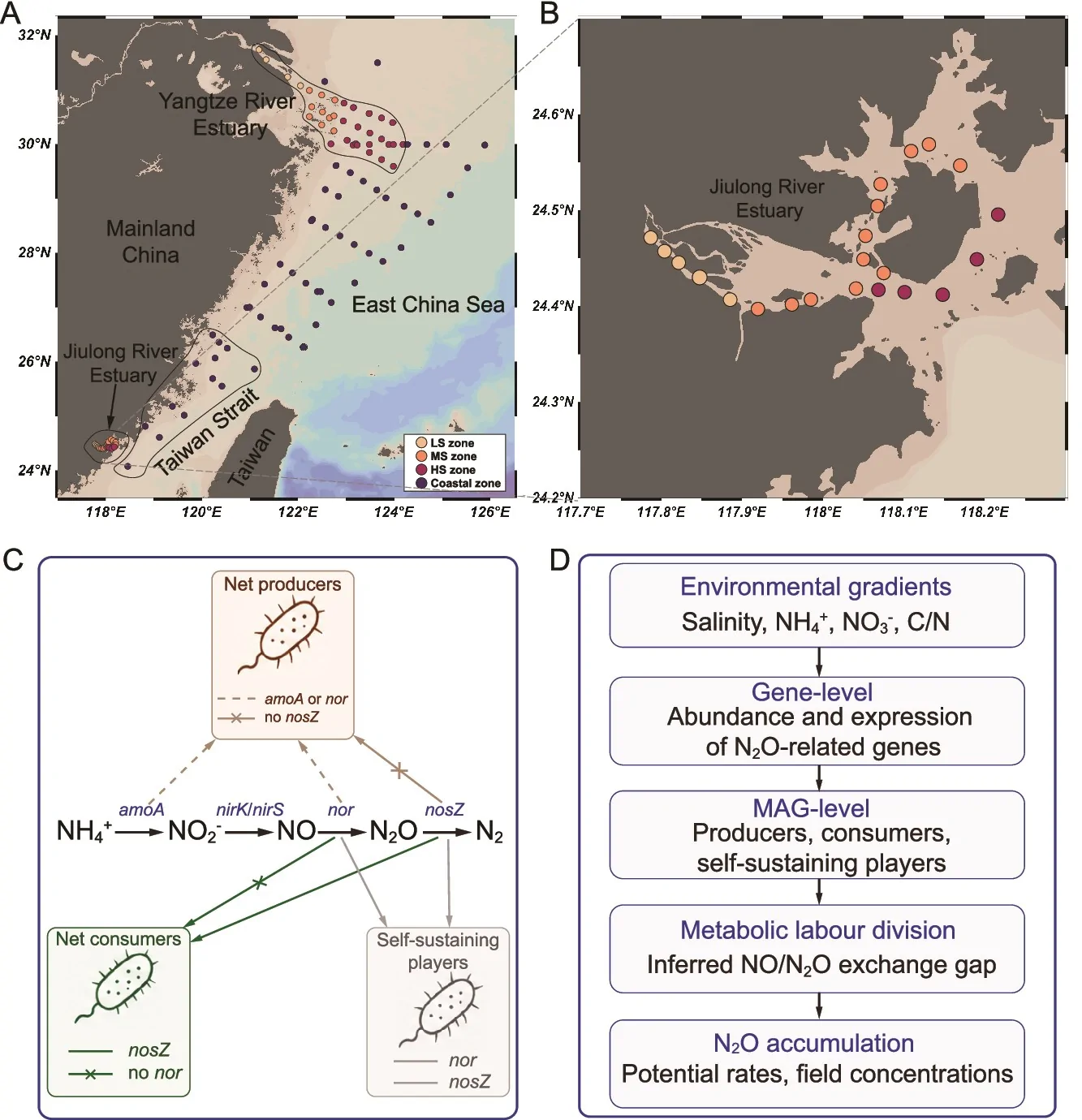
Study area, sampling design, and analytical framework. (A) Geographic distribution of sampling sites for measurements of N_2_O concentrations, environmental factors, metagenomes, and metatranscriptomes across YRE, ECS, TS, and JRE. (B) Sampling sites in the JRE where sediments were collected for metagenomes, metatranscriptomes, and N_2_O potential rate incubations. (C) Definition of three N_2_O-related microbial functional groups based on gene repertoires. (D) Conceptual workflow linking estuarine-coastal environmental gradients to gene-level abundance and expression, MAG-level functional-group organization, inferred NO/N_2_O exchange gap, and N_2_O accumulation.

Sampling covered the Yangtze River Estuary (YRE), the JRE, the adjacent East China Sea (ECS), and the Taiwan Strait (TS) (Fig. 1A and B), spanning river-influenced estuarine sediments to high-salinity coastal sediments. Detailed descriptions of the study regions are provided in Supplementary Methods.

A total of 120 surface (1 m below sea surface) and 86 bottom (~1 m above sediments) seawater samples were collected for dissolved N_2_O concentration measurements from the YRE (*n* = 64, July and October 2023), ECS (*n* = 66, October 2023), and JRE (*n* = 76, August and November 2023), following standard gas sampling protocols [21]. Each 100 ml sample was transferred into a pre-evacuated 200 ml aluminum foil gas sampling bag, preserved with 50 μl of 0.05% mercuric chloride, stored at room temperature, and analyzed within 1 month.

A total of 165 sediment samples were retrieved using a shipboard Van Veen grab sampler. The upper 0–5 cm layer was subsampled from the central, visually undisturbed portion of each grab using sterile 50 ml centrifuge tubes. These samples included 28 samples from the YRE (collected in October 2023), 47 samples from the ECS (collected in April and October 2023), 12 samples from TS (collected in July 2021), and 78 samples from the JRE (sampled across April, July, and November 2022, as well as February, May, August, and November 2023). All sediment samples were preserved at −80°C for subsequent metagenomic and metatranscriptomic sequencing. After DNA/RNA extraction, the remaining samples were freeze-dried for biogeochemical parameter analysis. Among these sediment samples, 48 had corresponding bottom-water N_2_O concentration measurements as well as other environmental and geochemical measurements. To expand the genome-resolved survey of N_2_O-related microbial groups across the same estuarine-coastal region, we also incorporated 117 previously collected samples. The full set of 165 samples was used for broad screening of N_2_O-related genes, metagenome-assembled genomes (MAGs), and functional groups, whereas correlation and path model analyses involving N_2_O concentrations were restricted to the 48 stations with complete matched environmental and metagenomic data.

Sediment samples used for N_2_O rate incubation were collected in July 2024 from 10 stations along the JRE because its accessibility enabled dense spatial sampling and same-day transport of samples to the laboratory for incubation and rate measurements. The 10 stations covered low-salinity (0–16 psu: stations A6, A7, A8), mid-salinity (16–32 psu: stations A9, X4, X5, X6), and high-salinity (>32 psu: stations JY2, X1, X2) zones. Samples were stored at 4°C and used for measurements of potential N_2_O production and consumption rates.

### Measurements of N_2_O concentrations and other environmental parameters

Dissolved N_2_O concentrations were quantified using the headspace equilibration method [21], and the detailed procedures are provided in the Supplementary Methods. Sediment sampling depths and overlying water temperatures were recorded by a conductivity-temperature-depth profiler (SBE 911, Sea-Bird Scientific). Sediment samples were homogenized with deionized water at a 1:2.5 ratio, and analyzed for salinity using a YSI Model 30 salinometer and pH values with a Fisher Scientific Star A211 pH meter equipped with an Orion 8157BNUMD combination electrode. Inorganic nitrogen species (NH_4_^+^, NO_2_^−^, NO_3_^−^) were extracted with 2 M KCl solution and quantified using a PowerMon Kolorimeter AA500 automated analyzer (Bran+Luebbe), and reported as μmol kg^−1^ dry sediment. Metal concentrations were determined by inductively coupled plasma mass spectrometry (ICP-MS; NexION 2000, PerkinElmer, USA) after sediment digestion with 1 M HCl [22]. Total organic carbon (TOC) and total nitrogen (TN) were measured with an elemental analyzer (Vario EL Cube, Elementar, Germany).

### Determination of potential N_2_O rates

Homogenized slurries were prepared by mixing sediments with overlying water at a 1:7 ratio (v:v) in 200 ml polycarbonate bottles, followed by 30-min helium purging to remove dissolved oxygen. A total of 3 ml slurry was transferred into each each 12 ml borosilicate vial (Labco Exetainers) and pre-incubated in the dark at 25°C for 48 h to consume any residual O_2_, NO_3_^−^, and NO_2_^−^. Two treatments were established: (i) ^15^NO_3_^−^ amendment (experimental group); (ii) ^15^NH_4_^+^ amendment which served as an anoxia control, in which ^45^N_2_O production was close to zero or slightly negative after background correction, indicating negligible nitrification-derived N_2_O production (Supplementary Table 1). Final ^15^N concentrations were adjusted to 75 μM. Spiked vials were incubated in the dark at *in situ* temperature. Incubation was terminated at 0, 2, 4, and 8 h by adding 50% ZnCl_2_. Isotopic species (^29^N_2_, ^30^N_2_, ^45^N_2_O, ^46^N_2_O) were quantified using a Thermo Finnigan Delta V Plus isotope ratio mass spectrometer. Each treatment was conducted with triplicate technical replicates per station. N_2_ and N_2_O production rates were calculated following previously published isotope-pairing methods [23, 24], and detailed calculation procedures are provided in Supplementary Methods.

### Metagenome sequencing and analysis

Detailed procedures for DNA extraction, library preparation, and sequencing are provided in the Supplementary Methods. Approximately 50 Gb of raw paired-end data were generated per metagenomic sample. Raw metagenomic reads were quality-filtered and adapter-trimmed with fastp (v0.23.2; default parameters) [25], and assembled into contigs using MEGAHIT (v1.2.9; default parameters) [26]. Only contigs >1000 bp were retained. Protein-coding genes were predicted using Prodigal (v2.6.3; -meta) [27] and clustered with CD-HIT (v4.8.1; -c 0.95 -aS 0.85) [28] to generate a non-redundant gene catalog containing 64 489 333 representative clusters. Functional annotation was performed using eggNOG-mapper (v2.1.9; default parameters) [29, 30].

MAGs were recovered using metabat2, maxbin2, and concoct within metaWRAP (v1.3.2) [31], supplemented by SemiBin2 (v2.1.0; single_easy_bin mode) [32] and VAMB (v4.1.3; --minfasta 200 000) [33]. MAGs from all tools were integrated and refined with the metaWRAP Bin_refinement module (v1.3.2; -c 50 -x 10) [31], with completeness and contamination assessed by CheckM (v1.2.1) [34]. MAGs were dereplicated at 95% average nucleotide identity (dRep, v3.4.3) [35] and screened for chimeras using GUNC (v1.0.5; default parameters) [36], yielding 2620 high- and medium-quality MAGs (completeness >=50%, contamination <10%). Relative abundances were determined with CoverM (v0.7.0; -m relative_abundance --trim-min 0.10 --trim-max 0.90 --min-read-percent-identity 0.95 --min-read-aligned-percent 0.75) [37], and taxonomy was assigned with GTDB-Tk (v2.7.2) against the GTDB R232 database [38]. The MAGs were functionally annotated with DRAM (v1.5.0; default settings) [39].

### Metatranscriptome sequencing and analysis

Metatranscriptomic analysis was conducted on 35 sediment samples from the YRE and adjacent ECS (*n* = 11) and the JRE (*n* = 24). Detailed procedures for RNA extraction, library preparation, and sequencing are provided in the Supplementary Methods. Approximately 50 Gb of raw paired-end data were generated for each metatranscriptomic sample. Raw reads were quality-controlled and adapter-trimmed with fastp (v0.23.2; default parameters) [25]. rRNA was removed using SortMeRNA (v4.3.6; default parameters) [40]. The remaining non-rRNA clean reads were subsequently used for gene quantification.

### Identification, validation, and quantification of N_2_O-related genes

*Nor* genes (including *cnor, qnor, enor, gnor, snor*, and *nnor*) were identified using reference sequences and hidden Markov models (HMMs) from the previously established database [13]. Targeted search for each *nor* variant across the 2620 MAGs and the non-redundant gene catalog was performed with two complementary approaches: DIAMOND (v0.9.14.115) in blastp mode (--evalue 1e-5) [41] and hmmsearch (-E 1e-5) in HMMER (v3.3.2) (http://hmmer.org/). All candidate *nor* genes detected by these methods were merged, and sequences <350 amino acids were removed. For the identification of *amoA, nosZ, nirK*, and *nirS*, we first queried protein sequences from the 2620 MAGs and the non-redundant gene catalog against NCycDB [42] and Greening lab metabolic marker gene databases [43] using DIAMOND (v0.9.14.115) blastp (-evalue 1e-5) [41]. The HMMs of bacterial *amoA*, archaeal *amoA, nosZ* clade I, *nosZ* clade II, and *nirK* were obtained from NCBI’s Protein Family Models or pfam database using accession NF041557.1, PF12942, TIGR04244.1, TIGR04246.1, and TIGR02376.1, respectively. The HMM of *nirS* was built by hmmbuild in HMMER (v3.3.2) using reference protein sequences (*n* = 438) downloaded from NCBI. These HMMs were used to screen proteins from the 2620 MAGs and non-redundant gene catalog with hmmsearch (-E 1e-5) in HMMER (v3.3.2). For *nosZ* clade III, reference sequences and the corresponding profile HMM(s) were taken from a published *nosZ* clade III study [16] and the associated repository. Candidate hits detected by blastp and HMMER were then validated using clade III-specific motifs [16]. Following the removal of truncated sequences, genes identified by both methods were merged as well.

Protein sequences identified by blastp and HMM searches were rigorously validated against the NCBI Conserved Domain Database [44] to confirm the presence of functionally relevant conserved domains. Phylogenetic trees were reconstructed using filtered amino acid sequences to verify their evolutionary affiliations relative to reference sequences. Multiple sequence alignments were performed using MAFFT (v7.525) [45], followed by automated trimming with TrimAL (v1.5.rev0; default parameters) [46]. Maximum-likelihood phylogenetic trees were generated with FastTree (v2.2.0.3, -lg) [47]. In parallel, protein tertiary structures were predicted using ESMFold [48], with reference structures obtained from AFDB [49] and PDB [50]. Structural phylogenies were constructed using Foldtree (https://github.com/DessimozLab/fold_tree) based on a local structural alphabet. Both phylogenetic and structural trees were visualized and annotated using the Interactive Tree of Life (iTOL; v6) [51]. Relative abundance and expression of N_2_O-related genes were quantified using Salmon (v1.10.3; --meta --validateMappings) [52]. Quantification results were normalized as genes per million (GPM) for metagenomic data and transcripts per million (TPM) for metatranscriptomic data.

### Genome-scale metabolic model analysis

GEMs were constructed only for high-quality N_2_O-associated MAGs using CarveMe (v1.6.4; default parameters) [53], with model quality evaluated with MEMOTE (v0.17.0; report snapshot mode) (https://github.com/opencobra/memote). The metabolic competition index (*MI_competition_*) and complementarity index (*MI_complementarity_*) between metabolic models were quantified by PhyloMInt (v0.1.0; default parameters) [54, 55]. *MI_competition_* and *MI_complementarity_* were regarded as the maximal metabolic competition and cooperation between pairwise MAGs, respectively [56]. NO and N_2_O exchange potentials were estimated using the “get_PTM.py” script (https://github.com/EmmettPeng/iNAP-PTM) [57], which implements metabolite transfer analysis based on metabolite annotations from CarveMe-reconstructed GEMs (BiGG Models database v1.6) [58]. Here, exchange potential indicates the model-inferred potential for metabolite exchange between microbial donors and recipients. For example, the NO exchange potential represents the potential transfer of NO from NO-producing populations to NO-reducing populations, whereas the N_2_O exchange potential represents the potential transfer of N_2_O from N_2_O-producing populations to N_2_O-reducing populations. The NO/N_2_O exchange gap, calculated as the NO exchange potential minus the N_2_O exchange potential, represents the imbalance between model-inferred upstream NO and downstream N_2_O exchange potentials within microbial communities.

### Definition of N_2_O-related functional groups and analytical roadmap

Based on the presence or absence of *amoA, nor*, and *nosZ*, N_2_O-related microorganisms were assigned to three functional groups. MAGs carrying *amoA* or *nor* but lacking *nosZ* were classified as net N_2_O producers, MAGs carrying *nosZ* but lacking *amoA* and *nor* were classified as net N_2_O consumers, and MAGs carrying both *nor* and *nosZ* were classified as self-sustaining N_2_O players (Fig. 1C). Although self-sustaining N_2_O players may still act as net producers or consumers based on *in situ* activity of *nor* and *nosZ*, this classification provided the basis for examining how environmental gradients were associated with gene abundance and expression, MAG-level functional-group composition, inferred metabolic division of labor, and N_2_O accumulation measured by field concentrations and potential rates (Fig. 1D). The distribution and salinity-related patterns of the three N_2_O functional groups were evaluated using both all MAGs and only high-quality N_2_O-associated MAGs (completeness >90% and contamination <5%).

### Statistical analysis

Differences in the abundance and expression of N_2_O-related genes among salinity zones were assessed using Wilcoxon rank-sum tests. Linear regressions were fitted using the lm function in R, and the significance of regression slopes was assessed from the fitted models. Multicollinearity among environmental variables was evaluated using variance inflation factors. Redundancy analysis, Mantel tests, Pearson correlations, and non-metric multidimensional scaling (NMDS) were used to evaluate relationships among environmental variables and functional groups. Partial least squares path modeling (PLS-PM) was performed using standardized variables in the R package plspm to evaluate predefined pathways linking environmental gradients, microbial functional groups, NO/N_2_O exchange metrics, and bottom-water N_2_O concentrations. Path coefficients were reported as standardized estimates, and model uncertainty was evaluated using 1000 bootstrap resampling. Effects were considered supported when the bootstrap confidence interval did not include zero. All R analyses were performed in R v4.4.0, and visualizations were generated using Chiplot (https://www.chiplot.online/).

## Results

### Low-salinity sediments show nutrient, organic-matter, and metal enrichment

**Figure 2:**
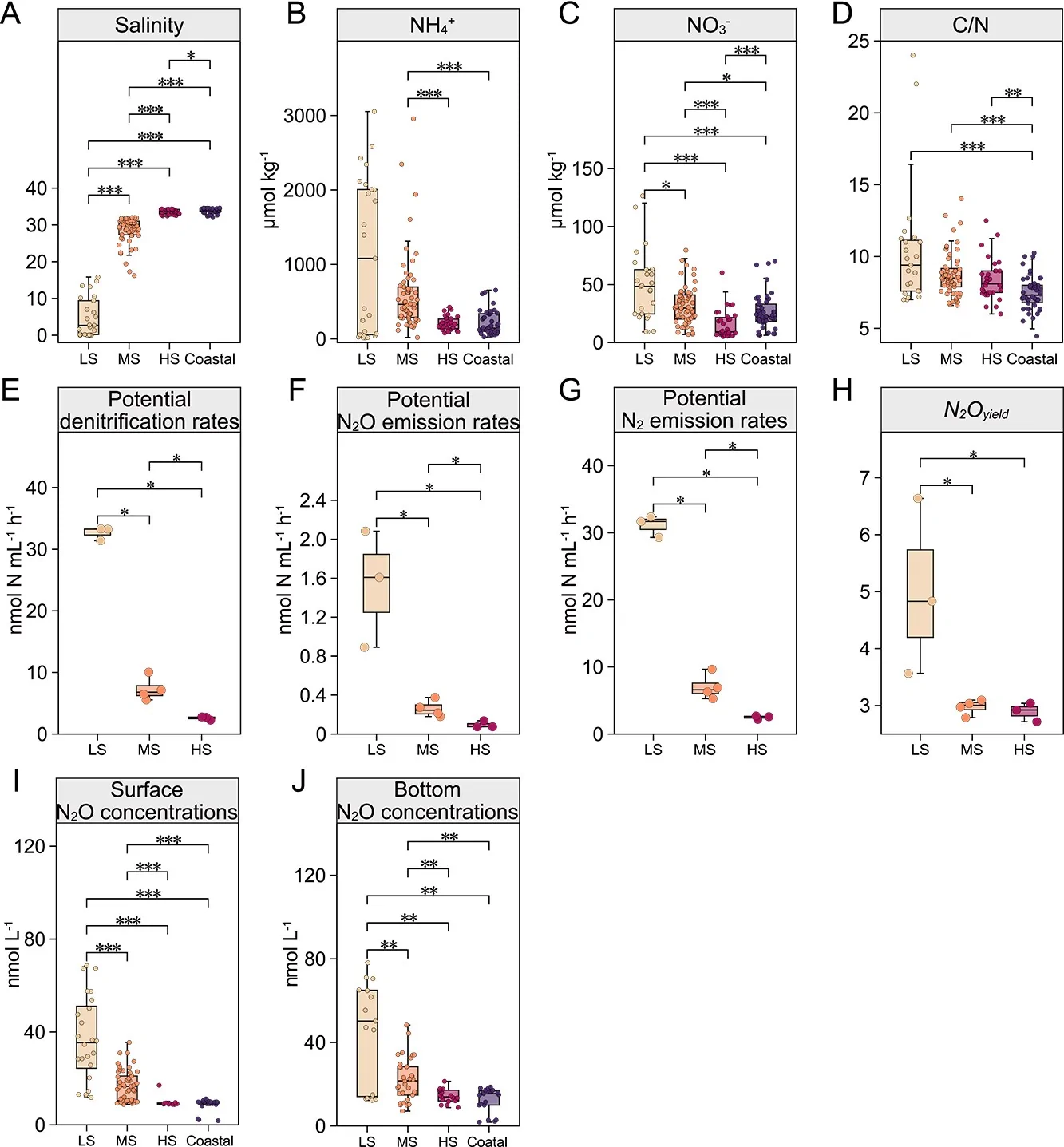
Environmental gradients, N_2_O-related potential rates, and N_2_O concentrations across estuarine-coastal zones. Comparison of (A) salinity, (B) NH_4_^+^, (C) NO_3_^−^, and (D) C/N among salinity zones. Sample sizes for environmental factors are LS (*n* = 21), MS (*n* = 53), HS (*n* = 27), and coastal (*n* = 64). (E) Potential denitrification rates (total NO_3_^−^ reduction to gaseous products, N_2_ + N_2_O), (F) potential N_2_O emission rates from denitrification (NO_3_^−^ reduction to N_2_O that accumulated rather than being further reduced), (G) potential N_2_ emission rates (NO_3_^−^ reduction to N_2_, approximately equal to N_2_O reduction), and (H) *N_2_O_yield_* of denitrification [N_2_O / (N_2_O + N_2_)] across LS (*n* = 3), MS (*n* = 4), and HS (*n* = 3) regions. (I) N_2_O concentrations in surface seawater across LS (*n* = 24), MS (*n* = 45), HS (*n* = 15), and coastal (*n* = 36) regions. (J) N_2_O concentrations in bottom water across LS (*n* = 15), MS (*n* = 33), HS (*n* = 14), and coastal (*n* = 24) regions. Statistical significance: ^*^P < .05, ^**^P < .01, ^***^P < .001.

Across the 165 sediment samples, salinity and sampling depth separated the dataset into four categories: low salinity (LS, *n* = 21, salinity: 0–16; average water depth: ~7 m), mid salinity (MS, *n* = 53, salinity: 16–32; average water depth: ~11 m), high salinity (HS, *n* = 27, salinity: >32; average water depth: ~44 m), and coastal zone (*n* = 64, salinity: >32; average water depth: ~68 m) (Fig. 1A and B, Fig. 2A, Supplementary Fig. 1A). Bottom-water temperature decreased from LS to coastal zones, whereas pH showed the opposite pattern (Supplementary Fig. 1B and C). Sediment inorganic nitrogen, organic matter, C/N ratio, and redox-active metals were generally enriched in LS sediments and declined toward coastal zones (Fig. 2B–D, Supplementary Fig. 1D–J). Overall, these environmental patterns indicate that the sampled sediments formed a clear estuary-to-coast gradient, with LS sediments providing a nutrient-, organic matter-, and metal-enriched setting that could support enhanced microbial N_2_O cycling.

### N_2_O production, yield, and concentrations peak in low-salinity zones

Using ^15^N isotope-labeling incubation, we quantified potential rates of denitrification (total NO_3_^−^ reduction to gaseous products, N_2_O + N_2_), N_2_O emissions from denitrification (NO_3_^−^ reduction to N_2_O that accumulated rather than being further reduced), and N_2_ emissions (NO_3_^−^ reduction to N_2_, approximately equal to N_2_O reduction) in surface sediments along the JRE salinity gradient. All three rates were significantly higher in LS and decreased seaward (*P* < .001) (Fig. 2E–G, Supplementary Fig. 2A–C). Specifically, potential denitrification rates ranged from 2.26 to 33.30 nmol N ml^−1^ h^−1^, accompanied by potential N_2_O emission rates of 0.07–2.08 nmol N ml^−1^ h^−1^ and potential N_2_ emission rates of 2.19–31.69 nmol N ml^−1^ h^−1^ (Supplementary Table 1). These rates were comparable to previous observations from nearby estuarine systems, such as the YRE and the Pearl River Estuary (PRE), where sediment N_2_O emission rates were ~0–10 nmol N ml^−1^ h^−1^ and N_2_O reduction rates were ~0–100 nmol N ml^−1^ h^−1^ [59]. The *N_2_O_yield_* of denitrification [N_2_O / (N_2_O + N_2_)] was generally higher in LS (averaging 5.0%) compared to MS (averaging 3.0%) and HS zones (averaging 2.9%) (Fig. 2H, Supplementary Table 1), suggesting a higher fraction of nitrogen oxides released as N_2_O at low salinity. Consistently, N_2_O concentrations in surface and bottom waters measured across the YRE, ECS, and JRE during summer (July to October 2023) were highest in LS (surface: 37.00 nmol/l; bottom: 45.09 nmol/l) and lowest in the coastal zone (surface: 9.07 nmol/l; bottom: 12.73 nmol/l) (Fig. 2I and J). N_2_O concentrations were significantly negatively correlated with salinity (surface: *R* = −0.71, *P* < .001; bottom: *R* = −0.67, *P* < .001) (Supplementary Fig. 3A and B). Together, these results revealed a spatial pattern of N_2_O dynamics that exhibits a maximum in the LS zone and declines seaward.

### Relative abundance and expression of N_2_O-related genes

**Figure 3:**
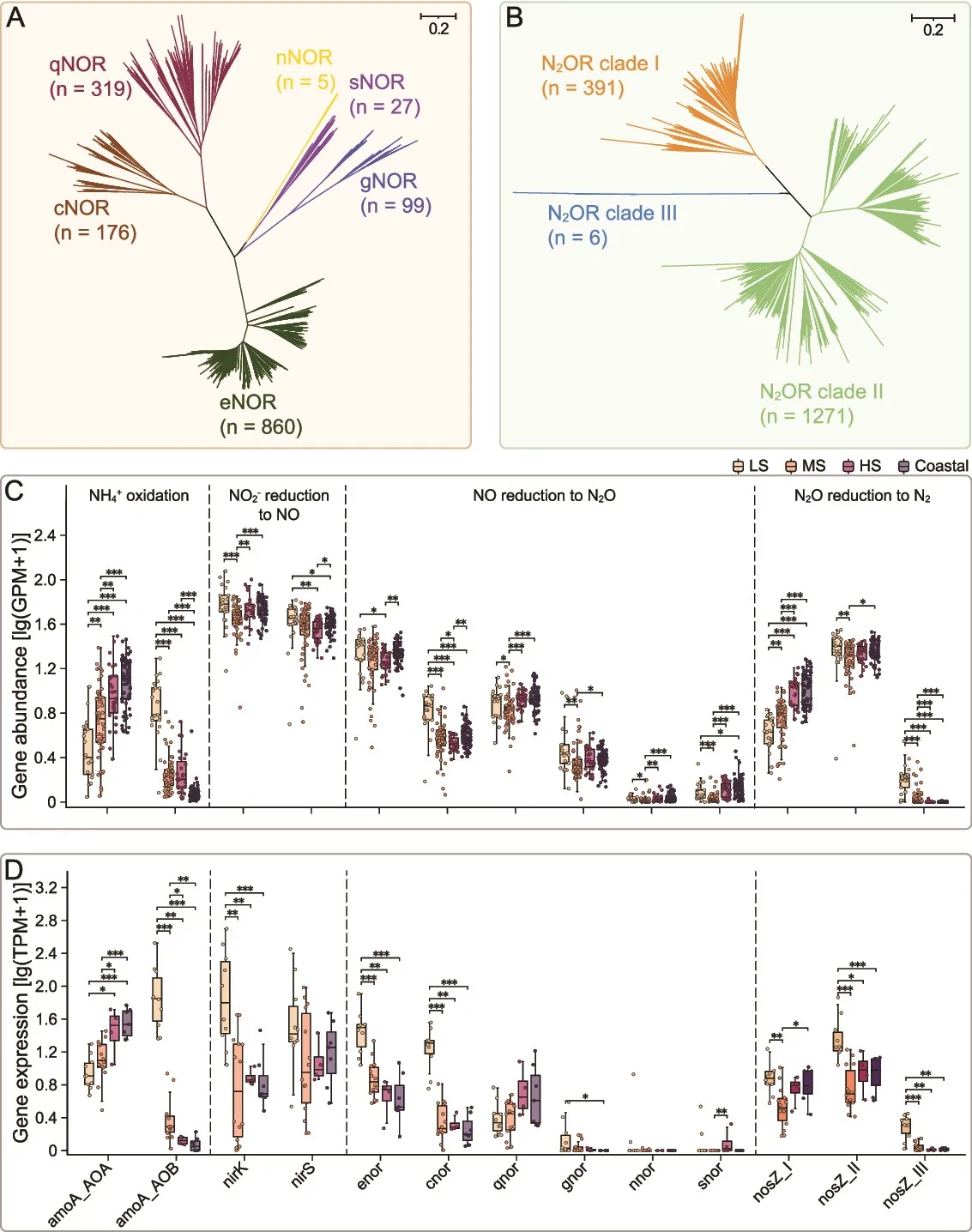
Identification and quantification of N_2_O-related genes. (A) Structural tree of the six clades of nitric oxide reductase (*nor*) proteins. (B) Structural tree of the three clades of nitrous oxide reductase (*nosZ*) proteins. Branch lengths represent structural distances. (C) Relative abundance of genes in metagenomes across LS (*n* = 21), MS (*n* = 53), HS (*n* = 27), and coastal (*n* = 64) regions. (D) Relative expression of genes in metatranscriptomes across LS (*n* = 11), MS (*n* = 14), HS (*n* = 4), and coastal (*n* = 6) regions. Statistical significance: ^*^P < .05, ^**^P < .01, ^***^P < .001.

From the non-redundant gene catalog containing 64 489 333 predicted genes across the sampling sites, we identified and validated N_2_O-related genes involved in nitrification and denitrification based on phylogenetic and structural analyses. These included 71 *amoA* genes (51 affiliated with AOA, *amoA_AOA*, and 20 affiliated with AOB, *amoA_AOB*) (Supplementary Fig. 4), 3032 *nirK* (Supplementary Fig. 5), 2387 *nirS* (Supplementary Fig. 6), 1486 *nor* (176 *cnor*, 319 *qnor*, 860 *enor*, 99 *gnor*, 27 *snor*, and 5 *nnor*) (Fig. 3A, Supplementary Fig. 7), and 1668 *nosZ* (391 clade I, 1271 clade II, and 6 clade III) (Fig. 3B, Supplementary Fig. 8).

The abundance and expression of *amoA_AOA* significantly increased with salinity (*P* < .001), whereas *amoA_AOB* showed the opposite pattern (Fig. 3C and D, Supplementary Fig. 9A–D), consistent with previously observed niche separation of AOA and AOB along estuarine gradients [60, 61]. For NO_2_^−^ reduction, *nirK* and *nirS* did not show significant correlation with salinity (Supplementary Fig. 10A and B), though both reached their highest average abundance in LS (Fig. 3C). At the transcript level, *nirK* showed a stronger negative correlation with salinity (*R* = −0.61, *P* < .001), whereas *nirS* showed a weaker negative correlation (*R* = −0.40, *P* < .05). *nirK* expression in LS was significantly higher than in other zones, whereas *nirS* expression did not differ significantly among salinity zones (Fig. 3D). For NO reduction, the combined abundance of all six *nor* clades exhibited a negative correlation with salinity (*R* = −0.46, *P* < .001) (Supplementary Fig. 11A), whereas the expression showed a strong negative correlation (*R* = −0.83, *P* < .001) (Supplementary Fig. 12A), with both metrics significantly higher in LS (*P* < .001, Supplementary Figs 11B and 12B). The abundance and expression of *cnor, enor*, and *gnor* genes significantly decreased with increasing salinity, whereas *qnor* exhibited a positive correlation with salinity (Fig. 3C and D, Supplementary Figs 11C–F and 12C–F). No significant patterns were observed for *snor* and *nnor* (Supplementary Fig. 11G and H). Among them, *enor* was the most abundant and also displayed the highest transcriptional activity. Although its biochemical turnover (~0.68 NO s^−1^ at 25°C) [13] is lower than NORs from mesophilic organisms such as *Pseudomonas stutzeri* (16 NO s^−1^) [62] or *Neisseria meningitidis* (30 NO s^−1^) [63], it exceeds activities of cNOR from thermophiles such as *Thermus thermophilus* (0.09 NO s^−1^) [64]. At the ecosystem level, eNOR might therefore make an important contribution to N_2_O production.

For N_2_O reduction, the combined *nosZ* abundance was higher in HS and coastal zones compared with the low-to-mid salinity regions (Supplementary Fig. 13A and B). Specifically, *nosZ* clade I showed a significant positive correlation with salinity (*P* < .001) (Fig. 3C, Supplementary Fig. 13C), clade II showed no clear salinity-related trend (Fig. 3C, Supplementary Fig. 13D), and clade III exhibited an opposite trend to clade I (*P* < .001) (Fig. 3C, Supplementary Fig. 13E). Metatranscriptomic data revealed significantly different expression dynamics: the combined *nosZ* expression and the respective expression of all three clades decreased significantly with increasing salinity (*P* < .001) (Supplementary Fig. 13F–J), with maximum expression observed in LS (Fig. 3D, Supplementary Fig. 13G).

### N_2_O-associated genomes separate into net producers, net consumers, and self-sustaining players

**Figure 4:**
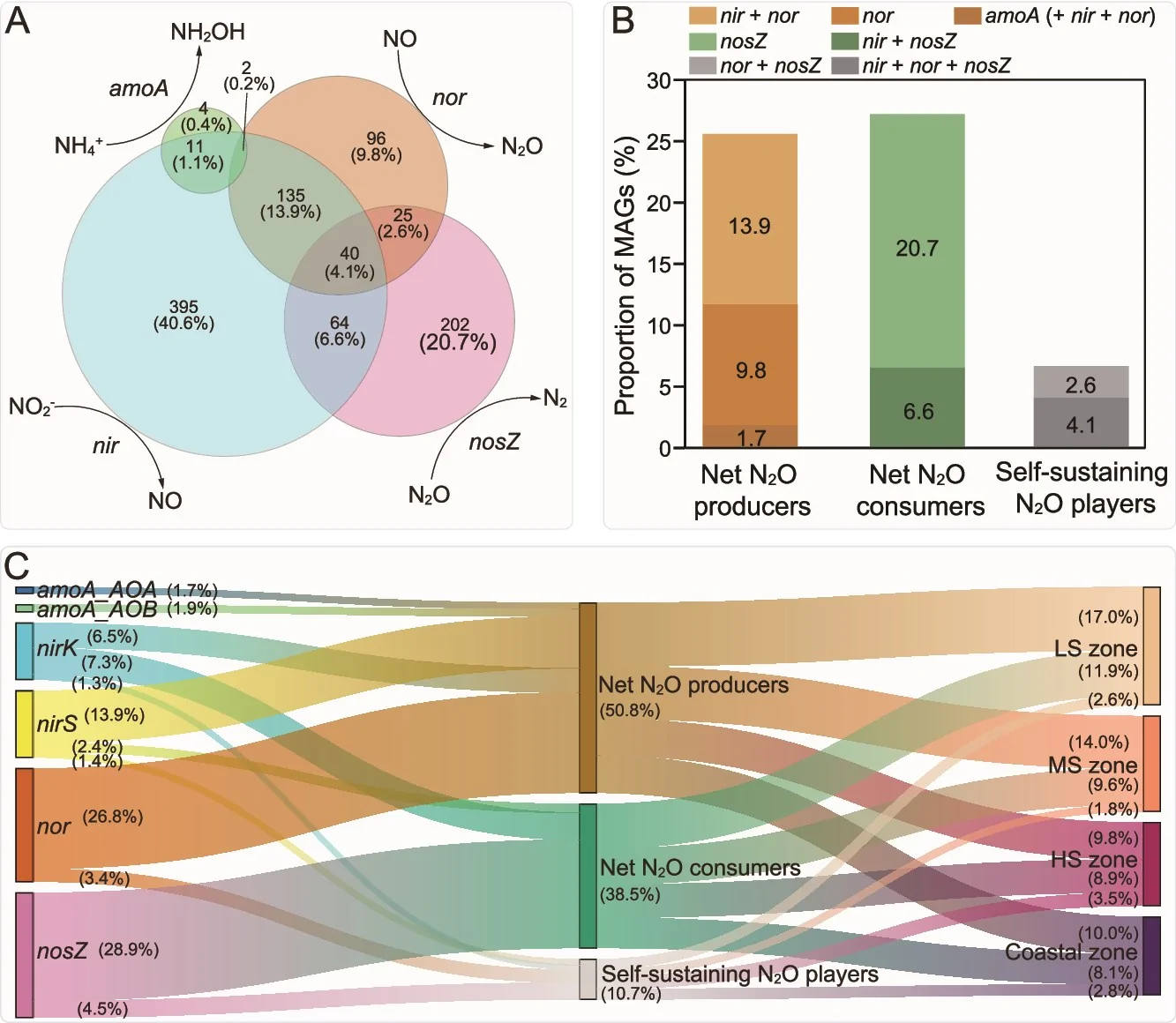
Classification and composition of N_2_O-related functional groups. (A) Area-proportional Euler diagram showing the MAG-count-based overlap among *amoA, nir, nor*, and *nosZ* gene carriage. The outer periphery of each circle indicates the enzymatic reactions catalyzed by the corresponding enzymes, and the area-proportional Euler diagram was scaled to the corresponding percentages of the total 974 N_2_O-associated MAGs. (B) The MAG-count-based classification of N_2_O-associated MAGs into net N_2_O producers, net N_2_O consumers, and self-sustaining N_2_O players. (C) Read-abundance-based distribution of N_2_O-associated MAGs from gene carriers to functional groups and salinity zones. Percentages were calculated from summed read-based relative abundance. Left-side values indicate the fraction of MAG abundance carrying each gene, middle values indicate the fraction of MAG abundance assigned to each functional group, and right-side values indicate the fraction of MAG abundance within each salinity zone.

A total of 974 MAGs with N_2_O-related genes were obtained (details are provided in Supplementary results, Supplementary Figs 14–18, and Supplementary Tables 2–6), including 9 MAGs with *amoA_AOB*, 8 MAGs with *amoA_AOA*, and 970 MAGs with denitrification genes (Fig. 4A). Among them, 599 MAGs (61.5%) were transcriptionally active (Supplementary Table 7), despite metatranscriptomic data being available for only ~20% of the metagenomic samples. Only 4.1% of MAGs encoded a complete denitrification pathway, whereas most represented incomplete denitrifiers (Fig. 4A).

Using the functional grouping scheme defined above, these MAGs were assigned to net N_2_O producers, net N_2_O consumers, or self-sustaining N_2_O players. On average, net N_2_O producers and net consumers accounted for similar proportions of the community (25.4% and 27.3%, respectively), whereas self-sustaining N_2_O players constituted a minor fraction (6.7%) (Fig. 4B). Net N_2_O producers were mainly composed of *nirK* (6.5% of all N_2_O-related MAG abundance), *nirS* (13.9%), and *nor* (26.8%) carriers, with minor inputs from *amoA_AOA* (1.7%) and *amoA_AOB* (1.9%), whereas net N_2_O consumers were dominated by *nosZ*-harboring taxa (28.9%) and self-sustaining N_2_O players included all gene groups (Fig. 4C).

Along the estuarine-coastal gradient, both net producers and net consumers were more abundant at low salinity, with their proportion peaking in LS and MS (for producers 17.0% and 14.0% of all N_2_O-related MAG abundance compared with ~10% in HS and coastal zones; for consumers 11.9% and 9.6% compared with 8.9% and 8.1%) (Fig. 4C). Self-sustaining N_2_O players were more evenly distributed and tended to be relatively enriched in HS and coastal sediments (3.5% and 2.8% versus 2.6% and 1.8% in LS and MS) (Fig. 4C). These spatial patterns were consistent with the correlation analyses, where the read-based relative abundance of net N_2_O producers and consumers decreased with salinity (producers *R* = −0.45, *P* < .001; consumers *R* = −0.38, *P* < .001) (Supplementary Fig. 19A and B) and was significantly higher in low-to-mid salinity zones than in HS and coastal zones (Supplementary Fig. 19D and E), whereas self-sustaining N_2_O players showed no significant correlation with salinity and tended to be more prevalent in HS and coastal zones (Supplementary Fig. 19C and F). The relative-abundance patterns of the functional groups based on high-quality MAGs were consistent with those based on all MAGs (Supplementary Fig. 20). At the metatranscriptomic level, the relative abundances of all three functional groups showed negative correlations with salinity (*R* = −0.55, −0.76, and −0.63, respectively, and *P* < .001 in all cases, Supplementary Fig. 21A, C, and  E). Their transcript-based relative abundances were generally highest in LS (Supplementary Fig. 21B, D, and  F). These patterns remained consistent when the analysis was restricted to high-quality MAGs (Supplementary Fig. 22). Thus, the distribution and activity of net N_2_O producers and consumers were consistent, whereas self-sustaining N_2_O players showed higher expression in metatranscriptomes in LS despite lower relative abundance in metagenomes.

### Changes in the composition of net N_2_O producers, consumers, and self-sustaining N_2_O players

**Figure 5:**
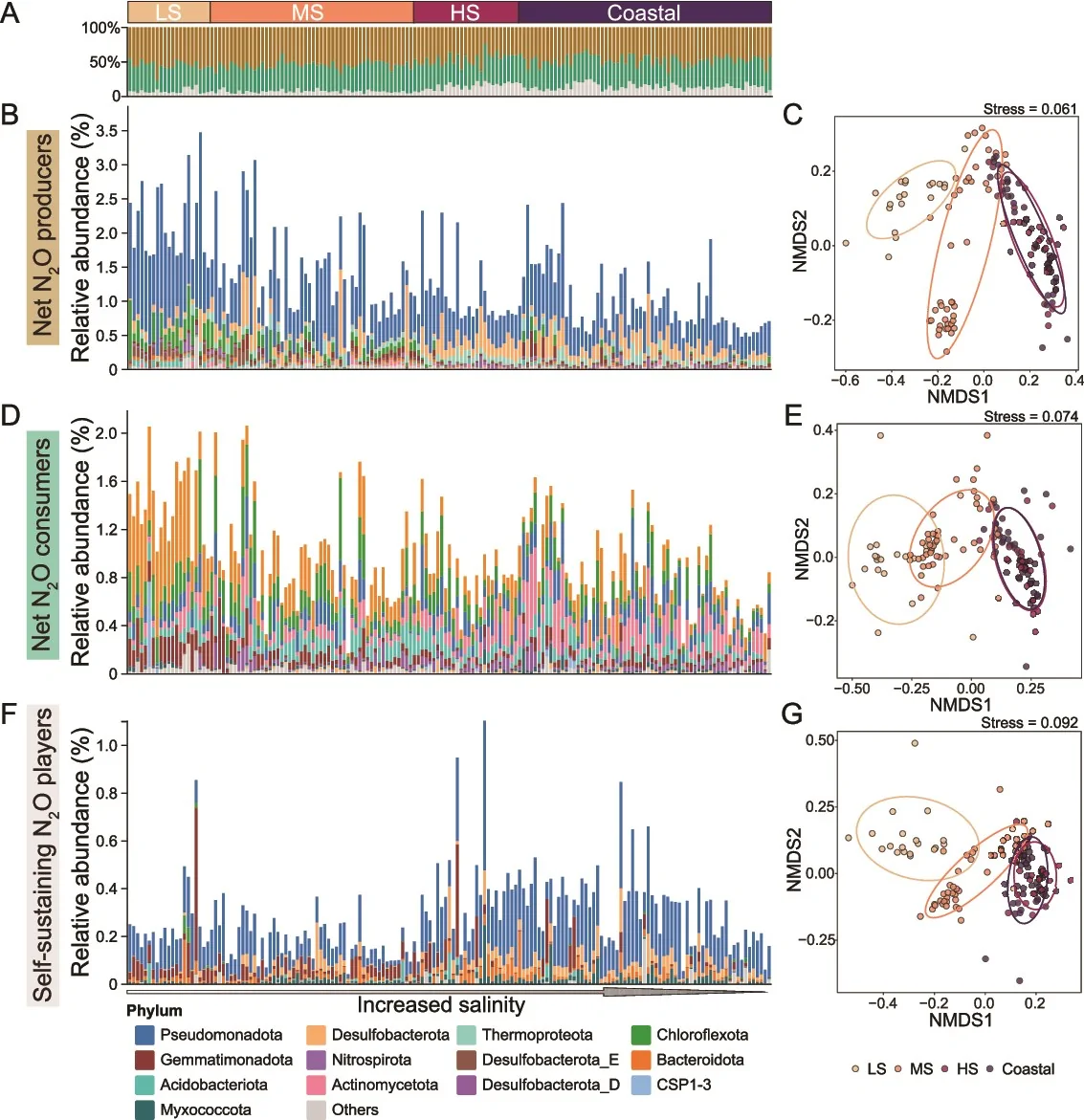
Shifts of N_2_O-related functional groups across the estuarine-coastal gradient. (A) Relative proportion of the three functional groups within the total N_2_O-associated microbial community. (B) Community composition and (C) NMDS analysis of the relative abundance of net N_2_O producers in 165 sediment samples. (D) Community composition and (E) NMDS analysis of the relative abundance of net N_2_O consumers. (F) Community composition and (G) NMDS analysis of the relative abundance of self-sustaining N_2_O players.

Among the three functional groups, net N_2_O producers and consumers were dominant in both metagenomes and metatranscriptomes, whereas self-sustaining N_2_O players accounted for a smaller proportion (Fig. 5A, Supplementary Fig. 23A). We next examined the taxonomic composition within each functional group to identify the lineages underlying these group-level patterns. Within the net N_2_O producers, *Woeseiales*-dominated *Pseudomonadota* was the most abundant phylum across all zones, and its relative abundance declined from 1.1% in LS to 0.63%–0.68% in HS and coastal zones (56.1% to 63.1% of all net producers) (Fig. 5B, Supplementary Fig. 24A, Supplementary Table 8). *Chloroflexota* was relatively enriched in LS (0.3% of the total community and 16.3% of all net N_2_O producers), whereas *Desulfobacterota* and *Thermoproteota* increased in relative abundance toward HS, reaching 0.24% and 0.09% of the total community (17.9% and 9.7% of all net producers), respectively (Fig. 5B, Supplementary Table 8). These major shifts were also observed in metatranscriptomes, where *Pseudomonadota* and *Chloroflexota* decreased in transcriptional activity along the estuarine-coastal gradient, whereas *Desulfobacterota* and *Thermoproteota* increased toward coastal zones (Supplementary Fig. 23B).

For net N_2_O consumers, *Bacteroidota* and *Gemmatimonadota* were more abundant at low salinity, decreasing from 0.4% and 0.2% of the total community in LS to 0.13% and 0.05% in coastal zones, respectively (Fig. 5D, Supplementary Table 9). These results corresponded to the decreases from 30.8% and 14.2% to 15.1% and 5.6% of all net N_2_O consumers (mainly driven by *Flavobacteriales* and *Gemmatimonadales*, respectively) (Supplementary Fig. 24B, Supplementary Table 9). In contrast, *Pseudomonadota* (with a more even composition) and *Actinomycetota* (dominated by *UBA5794*) became more abundant toward higher salinity, with their community-level abundance increasing from 0.10% and 0.08% in LS to 0.20% and 0.21% in HS (8.4% and 5.3% in LS to 19.7% and 20.9% in HS of all net N_2_O consumers, mainly driven by *UBA5794*), respectively (Fig. 5D, Supplementary Fig. 24B, Supplementary Table 9). The principal changes in the dominant consumer lineages were also evident in metatranscriptomes, with *Bacteroidota* and *Gemmatimonadota* decreasing and *Pseudomonadota* and *Actinomycetota* increasing in transcriptional activity across the estuarine-coastal gradient (Supplementary Fig. 23D).

Self-sustaining N_2_O players were consistently dominated by *Pseudomonadota* across all four salinity zones, accounting for 0.11%–0.25% of the total community (50%–67% of all self-sustaining N_2_O players, with dominance primarily from *Rhizobiales*) (Fig. 5F, Supplementary Fig. 24C, Supplementary Table 10). By contrast, the transcriptional activity of *Pseudomonadota* decreased along the estuarine-coastal gradient (Supplementary Fig. 21F). *Gemmatimonadota* was the second most abundant phylum in LS and MS (0.1% of the total community and 23%–29% of self-sustaining players), but its relative abundance decreased to <0.03% of the total community (<8% of all self-sustaining players) in HS and coastal regions (Fig. 5F, Supplementary Table 10), with a similar decline at the transcript level (Supplementary Fig. 23F).

NMDS analysis further revealed clear and coherent shifts in community structure across salinity zones for all three functional groups. Samples from LS and MS grouped separately from those in HS and coastal sediments, with stress values <0.1, indicating robust ordination performance (Fig. 5C, E, and  G). NMDS ordinations based on metatranscriptomic profiles showed a similar separation among salinity zones (Supplementary Fig. 23C, E, and  G), indicating that the salinity-associated shifts in community composition were also reflected at the transcript level.

### The NO/N_2_O exchange gap is associated with bottom-water N_2_O accumulation

**Figure 6:**
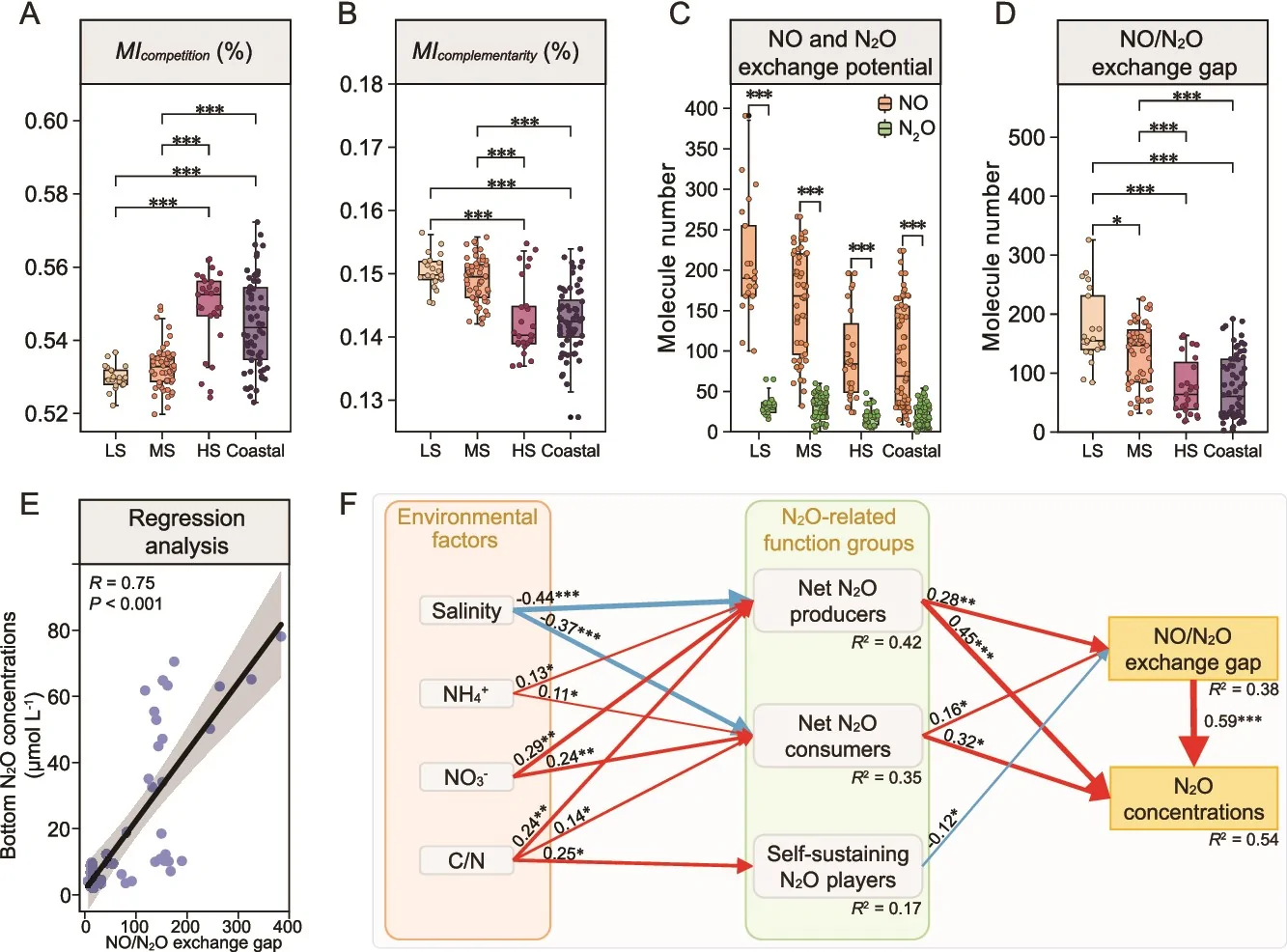
Salinity-associated metabolic interactions among N_2_O-related microorganisms and their associations with bottom-water N_2_O concentrations. (A–D) GEM-based estimates of metabolic competition (*MI_competition_*), metabolic complementarity (*MI_complementarity_*), NO and N_2_O exchange potentials, and NO/N_2_O exchange gap across LS (*n* = 21), MS (*n* = 53), HS (*n* = 27), and coastal (*n* = 64) regions. The NO/N_2_O exchange gap was calculated as the NO exchange potential minus the N_2_O exchange potential in the same sample. (E) Regression analysis between NO/N_2_O exchange gap and bottom-water N_2_O concentrations (*n* = 48). Shaded areas indicate 95% confidence intervals around the fitted regression lines. (F) PLS-PM showing the model-estimated direct effects of environmental factors, N_2_O-associated functional groups, and metabolite exchange on bottom-water N_2_O concentrations. Red and blue arrows indicate positive and negative effects, respectively, with effect direction also indicated by the signs of the standardized path coefficients next to the arrows. Arrow thickness is proportional to coefficient magnitude. *R*^2^ values indicate the variance explained for each endogenous variable. The overall goodness-of-fit of the model was 0.52 (*n* = 48). Statistical significance: ^*^P < .05, ^**^P < .01; ^***^P < .001.

GEMs were constructed for 229 high-quality N_2_O-associated MAGs (completeness >90%, contamination <5%), with total quality scores ranging from 68% to 88% (Supplementary Table 11). These GEMs were used to calculate pairwise metabolic interaction indices (*MI_competition_* for competition and *MI_complementarity_* for complementarity) in each metagenome. *MI_competition_* showed a positive correlation with salinity (*R* = 0.45, *P* < .001) (Supplementary Fig. 25A), reaching its maximum in HS (averaging 0.549) and minimum in LS (averaging 0.530) (Fig. 6A). In contrast, *MI_complementarity_* was negatively correlated with salinity (*R* = −0.46, *P* < .001) (Supplementary Fig. 25B), with the highest complementarity in LS (averaging 0.150) and the lowest in the coastal zone (averaging 0.143) (Fig. 6B). Together, these patterns suggested more metabolic cooperation and less competition within microbial communities in low-to-mid salinity zones compared to more coastal environments.

Because pathway fragmentation may involve microbial transfer of gaseous intermediates, we further quantified modeled NO and N_2_O exchange potentials across 165 metagenomes. NO exchange potential showed a negative correlation with salinity (*R* = −0.46, *P* < .001), whereas N_2_O exchange potential showed a weak but significant negative correlation (*R* = −0.27, *P* < .001) (Supplementary Fig. 25C). The NO/N_2_O exchange gap also exhibited a negative correlation with salinity (*R* = −0.48, *P* < .001) (Supplementary Fig. 25D). Across all four salinity zones, NO exchange potential was consistently higher than N_2_O exchange potential (*P* < .001) (Fig. 6C). The gap was greatest in LS, decreased with increasing salinity (Fig. 6D), and was significantly correlated with bottom-water N_2_O concentrations (*R* = 0.75, *P* < .001) (Fig. 6E).

Redundancy analysis and Mantel tests indicated that both N_2_O-associated functional genes and the three microbial functional groups were associated with salinity (Supplementary Figs 26 and 27). Because NH_4_^+^, NO_3_^−^, and the C/N ratio represent key substrates for nitrifying and denitrifying communities, these variables were used together with salinity in PLS-PM to explore how environmental factors were associated with microbial functional groups, NO/N_2_O exchange gap, and bottom-water N_2_O concentrations (Fig. 6F). The resulting model showed a satisfactory goodness-of-fit (0.52, *n* = 48), and bootstrap resampling (*n* = 1000) showed that the displayed structural paths had original coefficients close to the bootstrap means and 95% confidence intervals that did not include zero, indicating that the structural paths were stable (Supplementary Table 12). Salinity showed the highest and significant negative standardized path effects on both net N_2_O producers (β = −0.44, *P* < .001) and net N_2_O consumers (β = −0.37, *P* < .001). In comparison, NH_4_^+^ and NO_3_^−^ showed significant positive but minor effects on net N_2_O producers and consumers, and the C/N ratio was positively linked to all three functional groups. Together, these four environmental factors explained 42% of the variance in net N_2_O producers (*R*^2^ = 0.42), 35% in net N_2_O consumers (*R*^2^ = 0.35), and 17% in self-sustaining N_2_O players (*R*^2^ = 0.17). These three functional groups were associated with variation in the NO/N_2_O exchange gap, with positive effects from net N_2_O producers (β = 0.28, *P* < .01) and net N_2_O consumers (β = 0.16, *P* < .05), and a negative effect from self-sustaining N_2_O players (β = −0.12, *P* < .05). They together explained 38% of the variance in the NO/N_2_O exchange gap (*R*^2^ = 0.38). Among the variables included in the PLS-PM, N_2_O concentrations were most strongly associated with the NO/N_2_O exchange gap (β = 0.59, *P* < .001), with positive effects from net N_2_O producers (β = 0.45, *P* < .001) and net N_2_O consumers (β = 0.32, *P* < .05) as well. Together with environmental drivers (mainly indirect effects), all factors accounted for 54% of the variance in the observed bottom-water N_2_O concentrations. Overall, the model supported a statistical pathway in which environmental gradients reorganized N_2_O-related functional groups, and this reorganization was reflected in the NO/N_2_O exchange gap, which was in turn associated with N_2_O accumulation.

## Discussion

Although estuaries occupy only ~0.4% of the global ocean area, they are disproportionate hotspots of marine N_2_O emissions [8, 9]. Across the YRE, PRE, JRE, Scheldt Estuary, and other estuarine systems, dissolved N_2_O concentrations or saturation are generally highest in freshwater-influenced reaches and decline toward coastal waters [65–69]. Process-based studies have shown that denitrification (61.9%–81.3%) contributed more to N_2_O emissions than nitrification (18.7%–38.1%) in estuarine and coastal environments [70, 71]. Similar spatial patterns have been reported for N_2_O-related genes, with the abundance and transcription of *amoA, nirS, nirK*, and *nosZ*, as well as denitrifier community composition, varying with salinity, oxygen, and nitrogen availability across estuarine systems [72–77]. Consistent with this broader pattern, denitrification-associated potential rates, N_2_O concentrations, *N_2_O_yield_*, the transcription of *nor* and *nosZ*, and the net N_2_O producers and consumers were all highest in the LS zone and decreased seaward in our study, which established a clear spatial organization of N_2_O dynamics across the estuarine-coastal gradient.

The spatial pattern was likely associated with multiple covarying environmental factors across the estuarine-coastal transition. Elevated inorganic nitrogen and organic matter in LS sediments directly provide more substrates for nitrification and denitrification, explaining the higher N-transformation potential and the enrichment of N_2_O producers and consumers [7, 72, 73, 78]. Salinity represents an integrative estuarine environmental axis that covaries with sediment properties and other physicochemical conditions [7, 72, 79–83], showing the highest path effects on both net N_2_O producers and net N_2_O consumers (Fig. 6F). Fe and Cu may further affect metal-dependent denitrification enzymes [10, 13, 15], although their total concentrations do not resolve bioavailability or step-specific effects. Sediment oxygen profiles were not measured in this study, limiting our ability to evaluate the effects of oxygen availability on N_2_O cycling. Although oxygen penetration is typically restricted to the upper millimeters of coastal sediments, variation in penetration depth may alter the balance between nitrification and denitrification and, consequently, N_2_O production and consumption [82]. The variation in reduced sulfur compounds could additionally modify N_2_O production and reduction [84], but their contribution to the observed patterns cannot be resolved in this study. Overall, salinity-associated resource supply and physicochemical conditions provide the environmental basis for enhanced N_2_O cycling in LS sediments.

Genome-resolved analyses revealed that incomplete denitrifiers overwhelmingly dominated the N_2_O-associated communities, consistent with the widespread occurrence of partial denitrification pathways across natural and engineered microbiomes [17, 18]. The prevalence of *nor*-only microorganisms exemplifies this pathway fragmentation because these organisms can only reduce NO to N_2_O but require other community members to supply NO and reduce N_2_O [13, 17, 85]. The highest relative abundance in metatranscriptomes in LS sediments of net N_2_O producers, consumers, and self-sustaining players indicated greater functional partitioning, consistent with higher substrate availability supporting N_2_O-cycling activity [86]. Because self-sustaining players remained a minor fraction of the functional-group composition, their metagenomic pattern might be sensitive to shifts in the dominant producer and consumer groups, with their slight increase toward coastal sediments partly reflecting the stronger relative decline of producers and consumers [87]. The community composition of these functional groups also shifted. For example, the relative abundance of *Woeseiales* (*Pseudomonadota*) in net N_2_O producers and *Gemmatimonadota* in consumers and self-sustaining players declined from LS to coastal zones, consistent with previous observations of a negative association between *Woeseia* and salinity in estuarine sediments and the frequent occurrence of *Gemmatimonadota* in terrestrial and freshwater environments [88, 89]. These findings supported our first hypothesis that environmental gradients reorganize the relative abundance and composition of N_2_O-related functional groups. However, the concurrent enrichment of both N_2_O producers and consumers does not explain why N_2_O*_yield_* remained highest in LS sediments.

To link the functional group change to ecosystem-level behavior, we estimated NO and N_2_O exchange potentials and examined whether the imbalance between NO and N_2_O exchange potentials was associated with the *N_2_O_yield_* pattern. Each potential represents a donor-recipient opportunity for exchange of the corresponding metabolite [57]. In communities composed of partial denitrifiers, the NO exchange potential represents the potential transfer of NO from NO producers to NO consumers (N_2_O producers), and the N_2_O exchange potential represents the potential transfer of N_2_O from N_2_O producers to N_2_O consumers [17, 18, 90, 91]. The NO/N_2_O exchange gap therefore describes the imbalance between upstream NO exchange potential and downstream N_2_O exchange potential. As a disclaimer, this metric does not quantify realized N_2_O fluxes, but rather indicates potential imbalance in community-level metabolic coordination that could influence N_2_O accumulation. A larger positive gap in LS sediments indicated a stronger increase in potential NO exchange relative to potential N_2_O exchange. If these interactions are realized *in situ*, relatively greater routing of NO toward N_2_O producers than routing of N_2_O toward consumers could create a bottleneck, increasing the opportunity for N_2_O to accumulate in sediments or diffuse into bottom water [78, 90, 91]. Experimental evidence showing that N_2_O reduction by partial denitrifiers can depend on other denitrification intermediates supports the broader importance of community-level coordination in fragmented denitrification networks [91]. The correspondence of the exchange gap with measured *N_2_O_yield_*, together with its association with bottom-water N_2_O concentrations, is consistent with this bottleneck mechanism, which supported our second hypothesis. We therefore interpret the relationship between the exchange gap and N_2_O accumulation as a mechanistic hypothesis supported by convergent associations. Direct quantification of metabolite transfer and controlled experiments are needed to further validate this mechanism.

**Figure 7:**
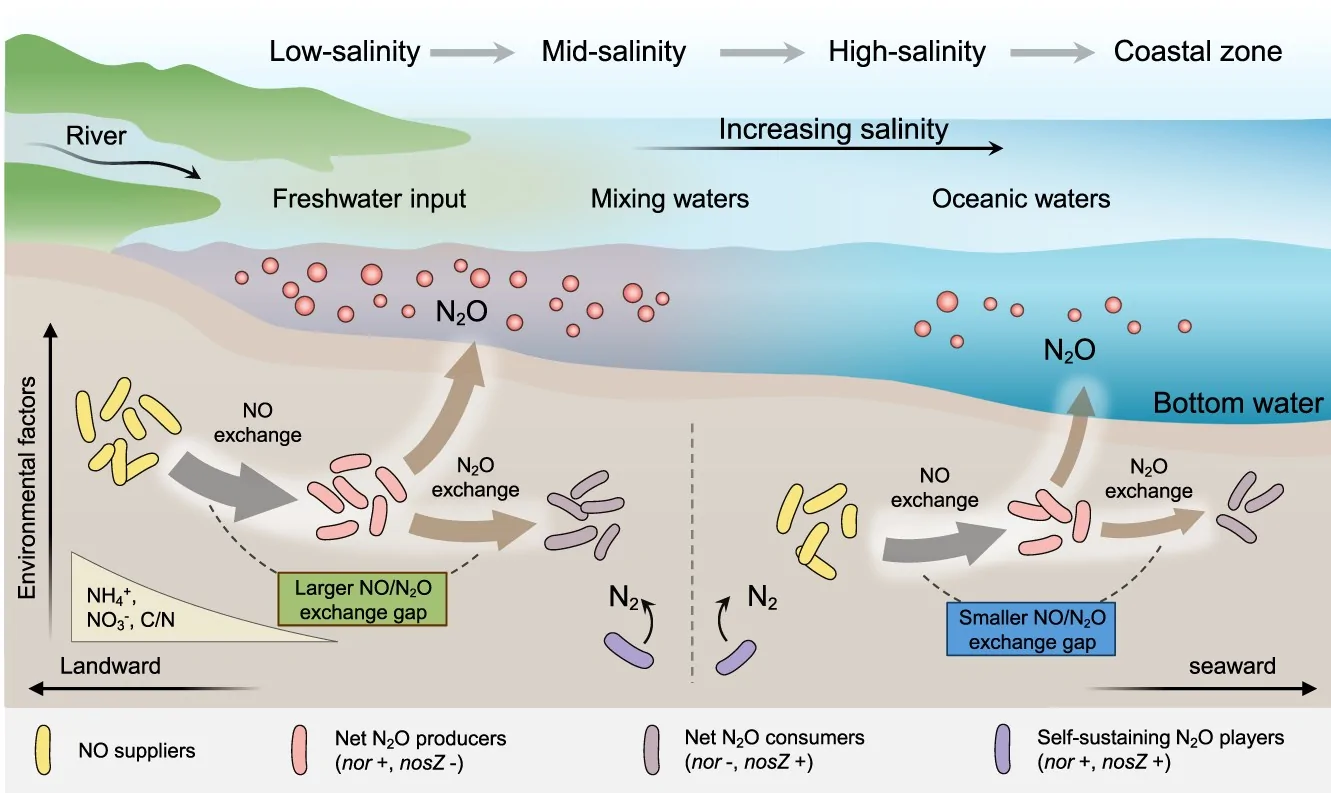
Metabolic division of labor drives N_2_O emissions in estuarine-coastal sediments. The organization of N_2_O-associated microbial functional groups is shaped along the estuarine-coastal gradient. LS sediments are characterized by a larger NO/N_2_O exchange gap, which is consistent with greater N_2_O accumulation and emission. In contrast, HS and coastal sediments show a relatively smaller gap. This framework identifies the NO/N_2_O exchange gap as a potential link between microbial community organization and bottom-water N_2_O concentrations.

Taken together, our results provided a framework with several ecological dimensions for understanding N_2_O accumulation across estuarine-coastal gradients (Fig. 7). First, environmental filtering associated with salinity and substrate availability reorganizes N_2_O-related microbial populations. Second, widespread pathway fragmentation creates the potential for community-level metabolic division of labor in which functional completeness emerges through interactions among incomplete denitrifiers. Third, the NO/N_2_O exchange gap is linked to the spatial pattern of N_2_O accumulation. This framework highlights a potential role for microbial community organization in shaping ecosystem-scale N_2_O dynamics. Future studies integrating isotope tracing, microscale spatial measurements, and controlled microbial community experiments will be needed to further validate the inferred metabolite handoffs and determine their contribution to *in situ* N_2_O fluxes.

## Supplementary Material

Supplementary_Materials_wrag216

## Data Availability

The raw sequencing data analyzed in this study are deposited in the NCBI under project accession numbers PRJNA1455734 and PRJNA1457255. Custom scripts used for data processing and statistical analyses have been deposited in Zenodo under DOI https://doi.org/10.5281/zenodo.21052207.
