# Supplementary material for: Metabolic division of labor drives estuarine-coastal N2O emissions
Source: ISME J. 2026 Aug 26;20(1):wrag216. doi: 10.1093/ismejo/wrag216 (PMC13592168; doi:10.1093/ismejo/wrag216)
Supplement: Supplementary file 1 [file supplementary_materials_wrag216.zip › Supplementary_Materials_wrag216.pdf]

Supplementary Information

Metabolic division of labour drives estuarine-coastal N_2O emissions

Enquan Zhang[1,2,7], Shengjie Li[3,7], Ehui Tan[4], Qiuyun Jiang[5], Yunxuan Li[1,2], Ziqi Wu[1,2], Jiaxin Chen[1,2], Xianhui Wan[1], Xianbiao Lin[6], Nengwang Chen[1],Yu Cong[1,2], Nianzhi Jiao[1,2], Xiyang Dong[5,*], Qiang Zheng[1,2,*]

[1]Department of Marine Biology and Technology, College of Ocean and Earth Sciences and State Key Laboratory of Marine Environmental Science, Xiamen University, Xiamen 361005, China.

[2]Fujian Key Laboratory of Marine Carbon Sequestration, Xiamen University, Xiamen 361005, China.

[3]State Key Laboratory of Submarine Geoscience, and School of Oceanography, Shanghai Jiao Tong University, Shanghai 200030, China.

[4]State Key Laboratory of Marine Resource Utilization in South China Sea, Hainan University, Haikou 570228, China.

[5]Key Laboratory of Marine Genetic Resources, Third Institute of Oceanography, Ministry of Natural Resources, Xiamen 361005, China.

[6]Frontiers Science Center for Deep Ocean Multispheres and Earth System and Key Laboratory of Marine Chemistry Theory and Technology, Ministry of Education, Ocean University of China, Qingdao 266100, China.

[7]These authors contributed equally: Enquan Zhang, Shengjie Li.

Pages: 34

Figures: 27

Tables: 12 (separate file)

Supplementary Methods

Study area

The Yangtze River, the third-longest river in the world, discharges into the East China Sea (ECS) near Chongming Island (31 °N, 121 °E) in Shanghai, forming the Yangtze River estuary (YRE), which is characterized by a subtropical monsoonal climate [1]. The ECS covers an area of 770,000 km^2, with an average water depth of 349 m and annual sea surface temperatures ranging from 20 to 24 °C. Intensified anthropogenic activities have led to severe nitrogen pollution in this system, with total annual nitrogen inputs estimated at 14.2 Tg, of which approximately 5.90 Tg are discharged into the Pacific Ocean [2]. Dissolved inorganic nitrogen concentrations in surface waters have increased from 0.5 mg L^{-1} in the 1960s to >1.8 mg L^{-1} in recent years [2]. The Jiulong River estuary (JRE), the second-largest river system in Fujian Province, is situated on the western coast of Xiamen Island, a densely populated and industrialized subtropical region. Its coastal waters receive freshwater inputs from the Jiulong River and are influenced by the China Coastal Current [3]. The JRE also experiences a subtropical monsoonal climate.

Measurements of N_2O concentration

Upon return to the laboratory, 80 mL of high-purity N_2 was injected into the aluminum foil gas sampling bags containing 100 mL of water sample to create a headspace. The bags were agitated on an orbital shaker at 150 rpm for 30 min to facilitate N_2O gas-liquid exchange, and then allowed to equilibrate statically for at least 4 hours (typically overnight). The headspace gas was transferred into pre-evacuated 200 mL aluminum foil gas sampling bags using gas-tight syringes for subsequent analysis. Ambient air samples were simultaneously collected with syringes to determine background N_2O concentrations. All gas samples were analyzed using a gas chromatograph with an electron capture detector (GC-2014, Shimadzu, Kyoto, Japan).

Calculation of N_2O-related potential rates

The calculation of N_2O-related potential rates was performed as follows:

$F_N = 2 \times P_{46} \times (P_{45} + 2 \times P_{46})^{-1}$ (1)

$D_{29} = 2 \times P_{30} \times (1 - F_N) \times F_N^{-1}$ (2)

$D_{N2O} = 2 \times P_{46} \times F_N^{-1}$ (3)

$D_{N2} = D_{29} + 2 \times P_{30}$ (4)

$D_t = D_{29} + 2 \times P_{30} + 2P_{46} \times F_N^{-1}$ (5)

$N_2O_{yield} = D_{N2O} \times D_t^{-1}$ (6)

where F_N represents the fraction of ^{15}N within experimental systems. D_{29} (nmol N mL^{-1} h^{-1}) quantifies denitrification-derived $^{29}N_2$ production rates. D_{N2O} (nmol N mL^{-1} h^{-1}) and D_{N2} (nmol N mL^{-1} h^{-1}) correspond to N_2O emission and N_2 production rates from denitrification, respectively. D_{N2} is approximately equal to N_2O reduction rates in our experiment [4]. The total potential denitrification rate is denoted by D_t, and N_2O_{yield} reflects the ratio of N_2O emission rates to total denitrification rates. P_{29}, P_{30}, P_{45}, and P_{46} represent 8-hour production rates for $^{29}N_2$, $^{30}N_2$, $^{45}N_2O$, and $^{46}N_2O$, respectively.

DNA extraction, library construction, and sequencing for metagenomes

For each sediment sample, DNA was extracted using the FastDNA Spin Kit for Soil (MP Biomedicals, CA, USA), then its purity and concentration were assessed using a NanoDrop One spectrophotometer (Thermo Fisher Scientific, MA, USA). High-throughput sequencing library preparations were constructed following the manufacturer’s protocol. 200 ng genomic DNA was randomly fragmented by Covaris to an average size of 300–350 bp. The fragments were treated with End Prep Enzyme Mix for end repair, 5’ phosphorylation and 3’ adenylated, to add adaptors to both ends. Size selection of Adaptor-ligated DNA was then performed by DNA Cleanup beads. Each sample was then amplified by PCR for 8 cycles using P5 and P7 primers, with both primers carrying sequences which can anneal with flowcell to perform bridge PCR and P7 primer carrying a six-base index allowing for multiplexing. The PCR

products were cleaned up and validated using an Agilent 2100 Bioanalyzer. The qualified libraries were sequenced pair-end PE150 on a DNBSEQ-T7 System (MGI Tech Co., Ltd., Shenzhen, China). Each sediment DNA sample was extracted, library-prepared and sequenced independently, with no biological pooling among samples.

RNA extraction, library construction, and sequencing for metatranscriptomes

For each sediment sample, RNA was extracted using the HiPure Soil RNA Kit (Magen, Guangzhou, China), then its purity and concentration were assessed using a Nanodrop One spectrophotometer (Thermo Fisher Scientific, MA, USA). 300–500 ng of total RNA was used for rRNA depletion by hybridization with single-stranded DNA probes specific to rRNA. The rRNA-probe hybrids were then enzymatically digested to remove both rRNA and the single-stranded DNA probes, and the remaining RNA was purified using RNA Clean Beads. The rRNA-depleted RNA was subsequently fragmented with divalent cations, followed by first-strand cDNA synthesis using random primers. Second-strand synthesis, 5’-end phosphorylation, and 3’-end adenylation (A-tailing) were performed in a single-tube reaction, and sequencing adapters were ligated to both ends of the double-stranded cDNA. Adapter-ligated products were purified and size-selected using DNA Clean Beads. The libraries were then PCR-amplified with the addition of UDG enzyme to digest uracil-containing strands, using P5 and P7 primers for amplification. Library quality was assessed prior to sequencing. Finally, libraries with different index sequences were pooled and sequenced in paired-end mode (PE150) on a DNBSEQ-T7 System (MGI Tech Co., Ltd., Shenzhen, China) according to the manufacturers’ instructions. Each RNA sample was extracted, library-prepared and sequenced independently, with no biological pooling among samples.

Supplementary results

N_2O-related genes in metagenome-assembled genomes (MAGs) and their host lineages

Across the non-redundant genome catalog of 2,620 MAGs, we characterized the diversity and phylogenetic affiliations of key N_2O-related genes. 10 *amoA_AOB* and 8 *amoA_AOA* were identified based on phylogenetic (Supplementary Fig. 14A) and structural analyses (Supplementary Fig. 14B). The *amoA_AOB* sequences displayed higher phylogenetic diversity and were affiliated with *Pseudomonadota* (*n* = 5), *Chloroflexota* (*n* = 3), *Desulfobacterota* (*n* = 1), and *Nitrospirota* (*n* = 1), whereas all *amoA_AOA* were affiliated with *Thermoproteota* (Supplementary Fig. 14A, B, Supplementary Table 2). Among the recovered AOB MAGs, two genomes also harbored *nirK* and *nor* genes, with one encoding *enor* and the other *cnor*, suggesting the potential for nitrifier denitrification and consequent N_2O production. No Comammox genomes were detected in this study.

From the same MAG catalogue, 266 *nirS* (Supplementary Fig. 15A, B) and 544 *nirK* sequences (489 bacterial and 55 archaeal) (Supplementary Fig. 16A, B) were identified. *nirS* was detected in 13 bacterial phyla, dominated by *Gammaproteobacteria* (65.79%) and *Alphaproteobacteria* (17.67%) within *Pseudomonadota* (Supplementary Table 3). *nirK* showed a broader taxonomic distribution, spanning 23 bacterial and 4 archaeal phyla (Supplementary Table 4). Major bacterial carriers of *nirK* included *Pseudomonadota* (*n* = 138), *Chloroflexota* (*n* = 96), *Actinomycetota* (*n* = 74), *Gemmatimonadota* (*n* = 53), and *Nitrospirota* (*n* = 43), whereas archaeal *nirK* was mostly affiliated with *Thermoproteota* (*n* = 47) (Supplementary Table 4).

Phylogenetic and structural analyses identified 308 *nor* genes (Supplementary Fig. 17A, B). In addition to the well-characterized *cnor* (*n* = 50) and *qnor* (*n* = 44), we detected four additional clades: *enor* (*n* = 196), *gnor* (*n* = 11), *nnor* (*n* = 5), and *snor*

(*n* = 2) (Supplementary Fig. 17A, B). These *nor* genes were distributed across 19 bacterial phyla, with *Pseudomonadota* representing the most frequent hosts (*n* = 186), followed by *Armatimonadota*, CG2-30-53-67, *Deinococcota*, and *Desulfobacterota_B* (Supplementary Table 5). Several MAGs affiliated with *Pseudomonadota*, *Gemmatimonadota*, and *Desulfobacterota* encoded two distinct *nor* clades within a single genome (Supplementary Table 5), suggesting potential metabolic flexibility under fluctuating environmental conditions.

We also identified 339 *nosZ* sequences that were assigned to clades I (*n* = 68), II (*n* = 270), and III (*n* = 1) (Supplementary Fig. 18A, B). Across the 19 host phyla, clade I occurred in only five phyla and was dominated by *Pseudomonadota* (*Alpha*- and *Gammaproteobacteria*, 92.42%). The only clade III sequence also belonged to *Alphaproteobacteria* (Supplementary Table 6). In contrast, clade II showed the broadest phylogenetic range, spanning 18 phyla, with the most frequent hosts including *Bacteroidota* (30.83%), *Chloroflexota* (14.66%), *Actinomycetota* (13.16%), *Gemmatimonadota* (11.28%) and *Acidobacteriota* (10.15%) (Supplementary Table 6). Notably, *Myxococcota_A* was identified as a previously unrecognized *nosZ*-containing phylum with potential N_2O reduction capacity. Two MAGs, A7-7-2310_bin.2 (*Pseudomonadota*) and S01-2-2304_bin.1 (*Chloroflexota*), carried both clade I and clade II *nosZ* genes (Supplementary Table 6). Together, these N_2O-related genes included 13 functional gene clades distributed across 37 microbial phyla, showing their high diversity in estuarine-coastal sediments.

Supplementary figures

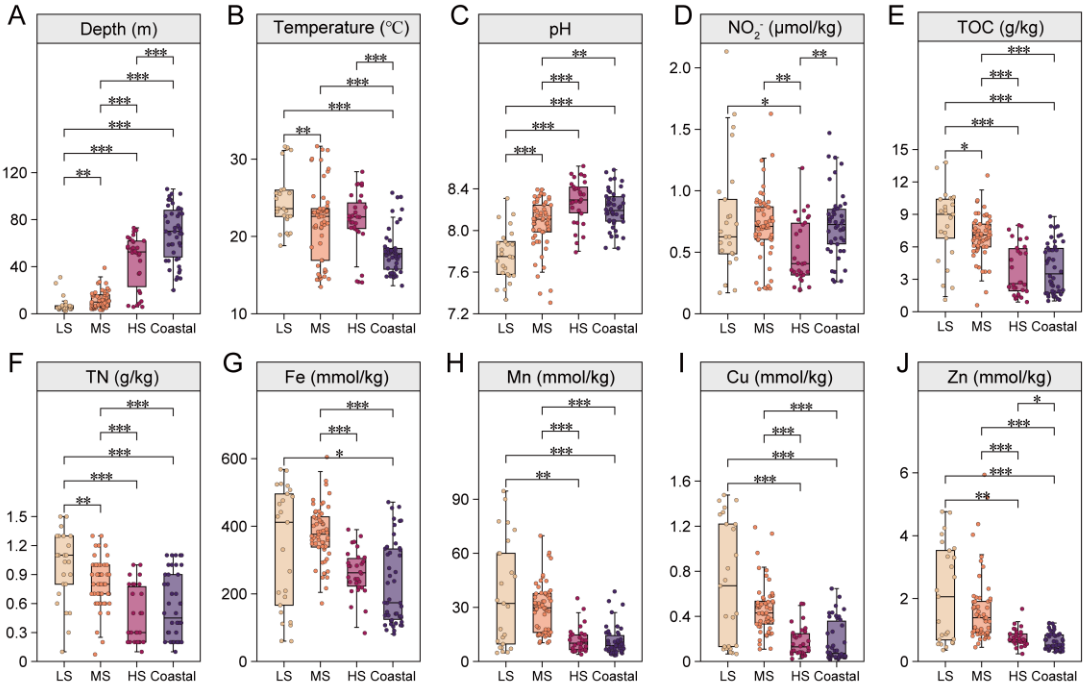

Supplementary Figure 1. Environmental factors across salinity zones. (**A**) Water depth. (**B**) Bottom temperature. (**C**) pH. (**D**) NO_2^-. (**E**) TOC. (**F**) TN. (**G**) Dissolved sediment Fe. (**H**) Dissolved sediment Mn. (**I**) Dissolved sediment Cu. (**J**) Dissolved sediment Zn. Sample sizes were identical across (**A**–**J**): LS (n = 21), MS (n = 53), HS (n = 27), and Coast (n = 64). Statistical significance: * $P < 0.05$; ** $P < 0.01$; *** $P < 0.001$.

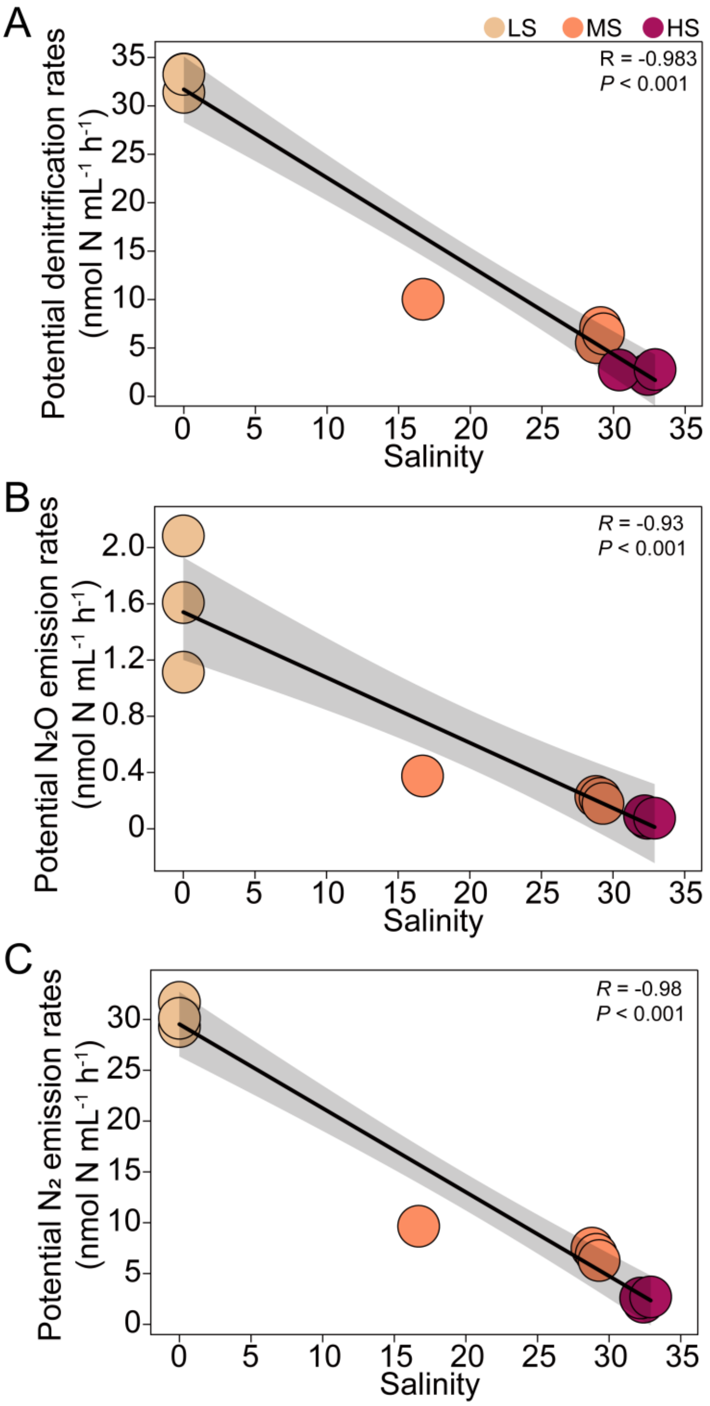

Supplementary Figure 2. N_2O-related potential rates determined by incubation experiments. Correlation between (**A**) potential denitrification rates, (**B**) N_2O emission rates, (**C**) N_2 emission rates and salinity, respectively (n = 10). Shaded areas indicate 95% confidence intervals around the fitted regression lines.

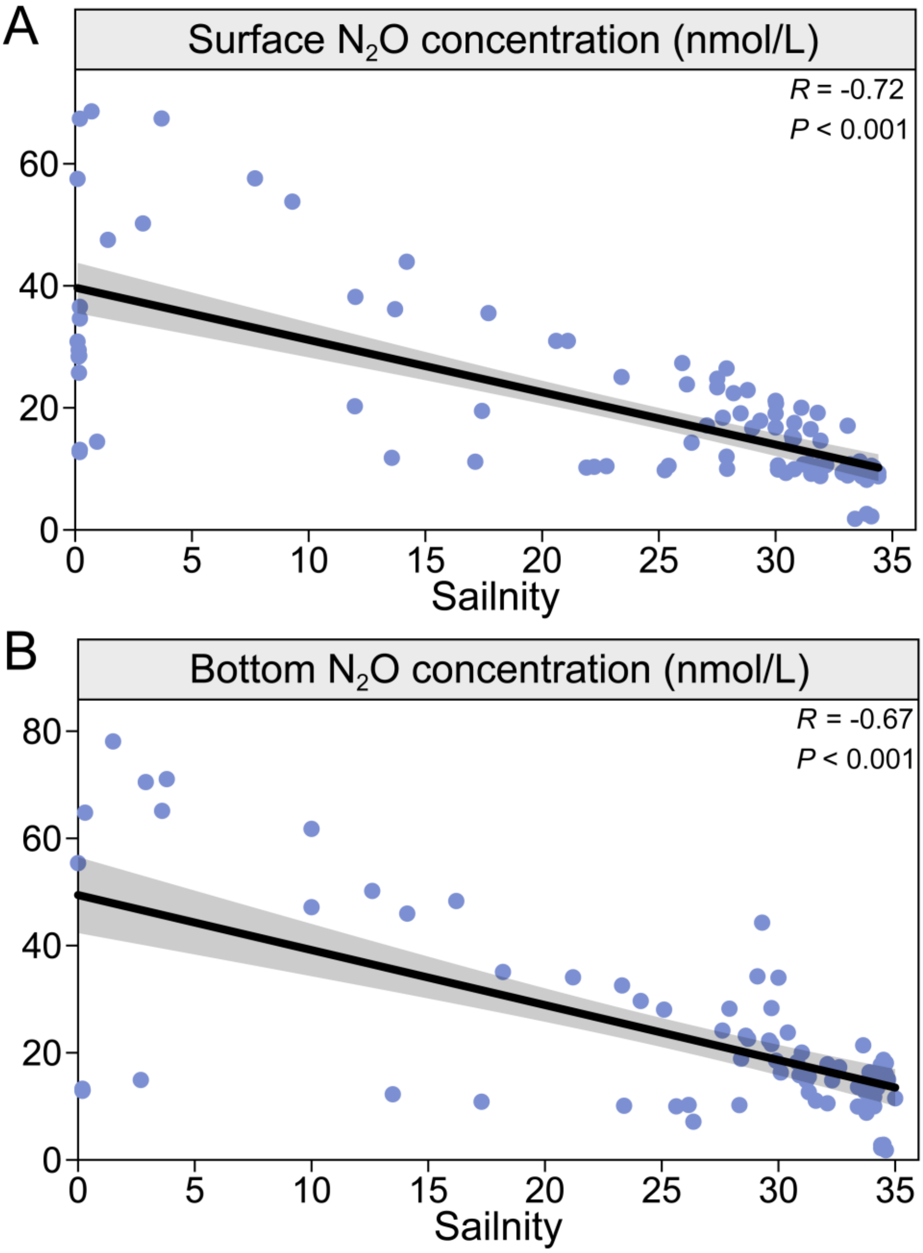

Supplementary Figure 3. Distribution of N_2O concentrations in seawater along with salinity gradient. (**A**) Surface seawater. (**B**) Bottom seawater. Shaded areas indicate 95% confidence intervals around the fitted regression lines.

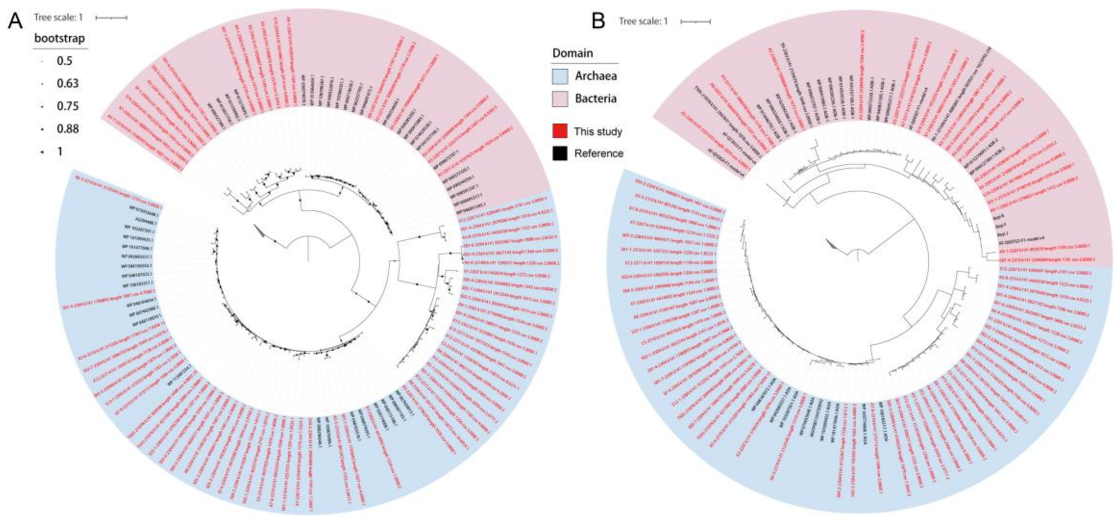

Supplementary Figure 4. Identification of *amoA* genes from the non-redundant gene catalog. (**A**) Maximum-likelihood phylogenetic tree of *amoA* genes. Bootstrap values >50% are shown. Scale bar indicates the mean number of substitutions per site. (**B)** Structural tree of *amoA* genes. Branch lengths represent structural distances.

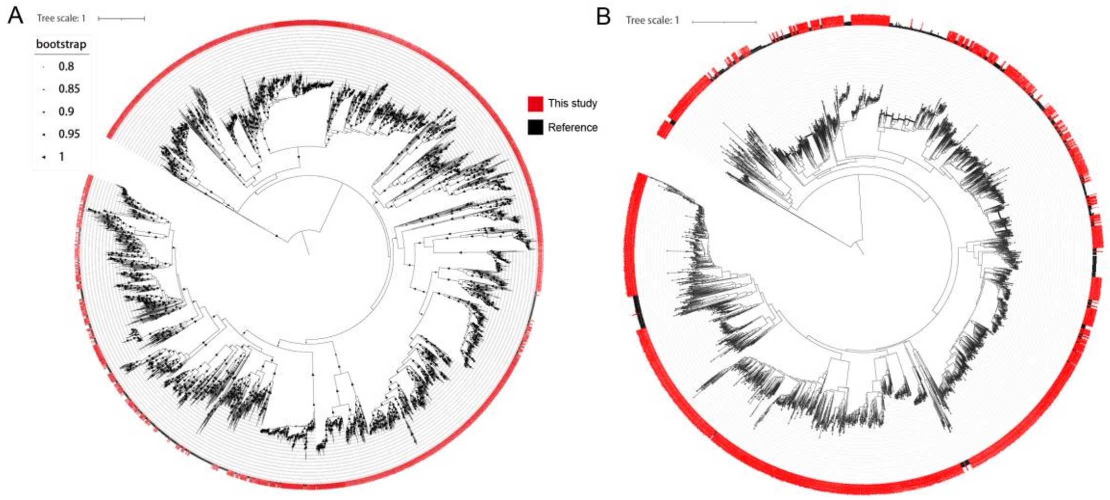

Supplementary Figure 5. Identification of *nirK* genes from the non-redundant gene catalog. (**A**) Maximum-likelihood phylogenetic tree of *nirK* genes. Bootstrap values >80% are shown. Scale bar indicates the mean number of substitutions per site. (**B**) Structural tree of *nirK* genes. Branch lengths represent structural distances.

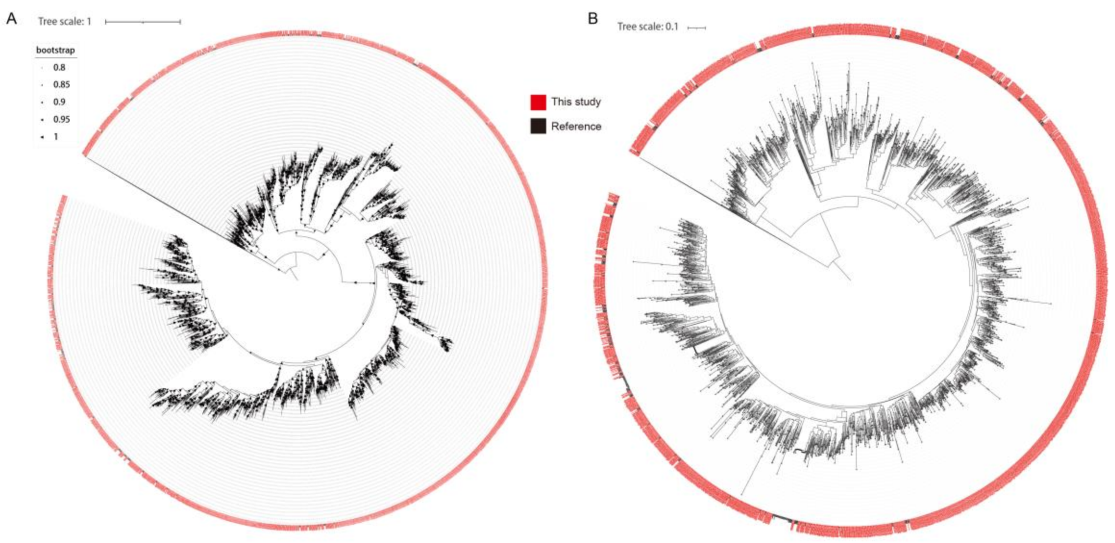

Supplementary Figure 6. Identification of *nirS* genes from the non-redundant gene catalog. (**A**) Maximum-likelihood phylogenetic tree of *nirS* genes. Bootstrap values >80% are shown. Scale bar indicates the mean number of substitutions per site. (**B**) Structural tree of *nirS* genes. Branch lengths represent structural distances.

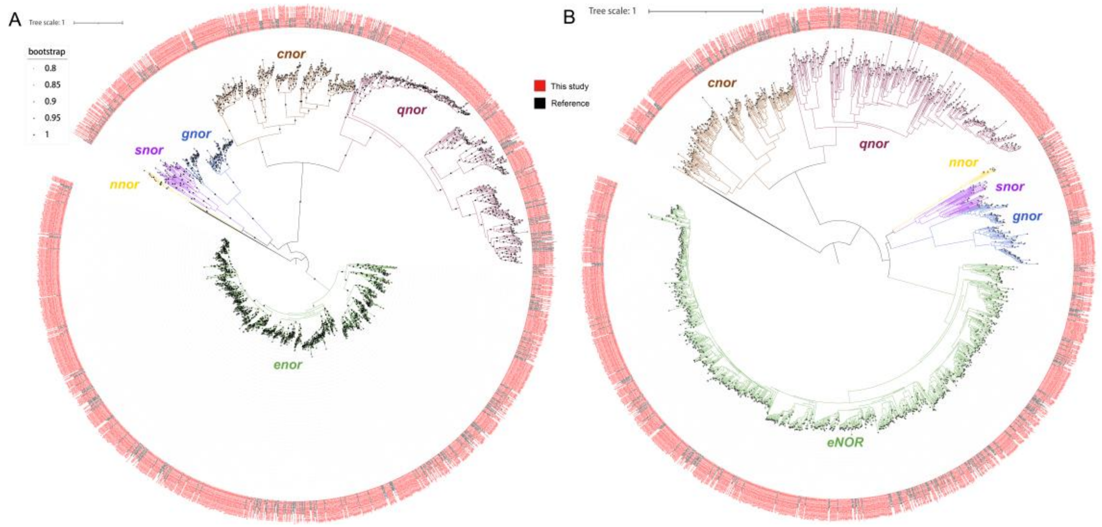

Supplementary Figure 7. Identification of *nor* genes from the non-redundant gene catalog. (**A**) Maximum-likelihood phylogenetic tree of *nor* genes. Bootstrap values >80% are shown. Scale bar indicates the mean number of substitutions per site. (**B**) Structural tree of *nor* genes. Branch lengths represent structural distances.

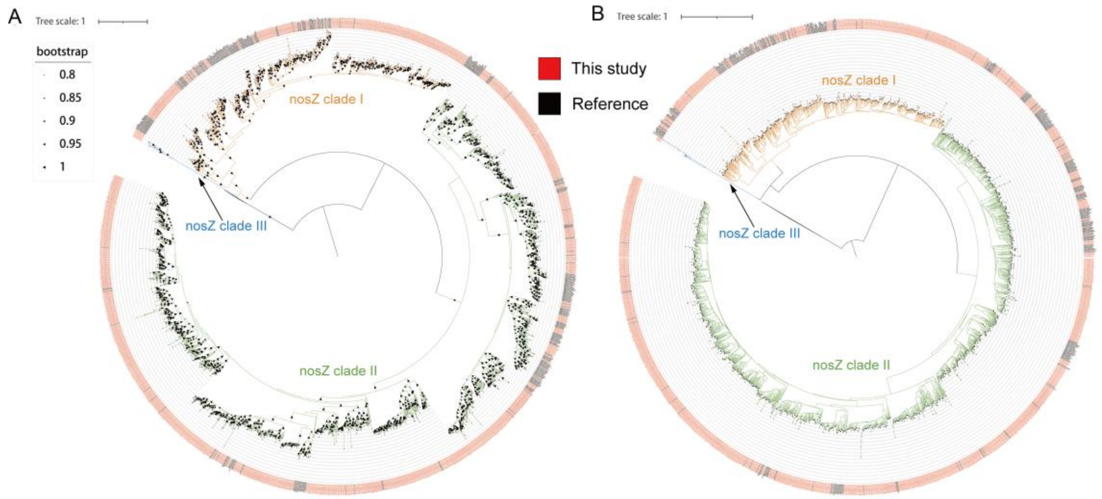

Supplementary Figure 8. Identification of *nosZ* genes from the non-redundant gene catalog. (**A**) Maximum-likelihood phylogenetic tree of *nosZ* genes. Bootstrap values >80% are shown. Scale bar indicates the mean number of substitutions per site. (**B**) Structural tree of *nosZ* genes. Branch lengths represent structural distances.

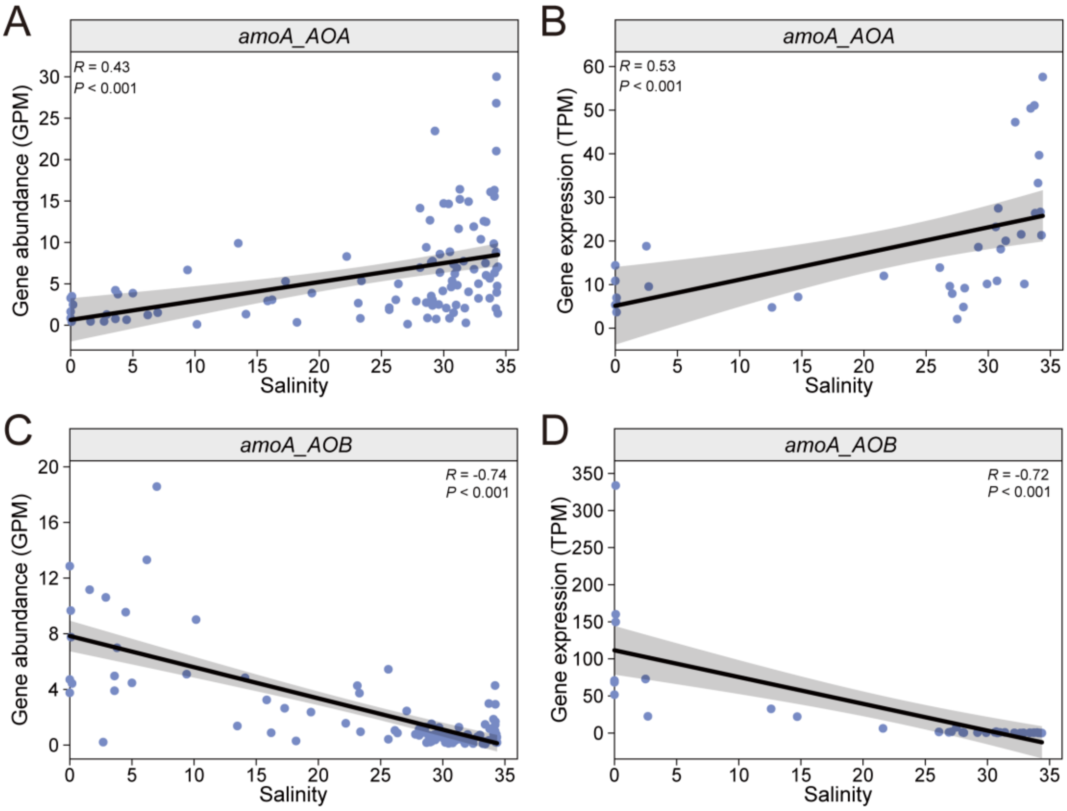

Supplementary Figure 9. Relative abundance and expression of *amoA* genes across salinity gradient. (**A**) Relative abundance and (**B**) expression of *amoA_AOA* along the salinity gradient. (**C–D**) Variations of the gene abundance and expression of *amoA_AOB* along the salinity gradient. Shaded areas indicate 95% confidence intervals around the fitted regression lines.

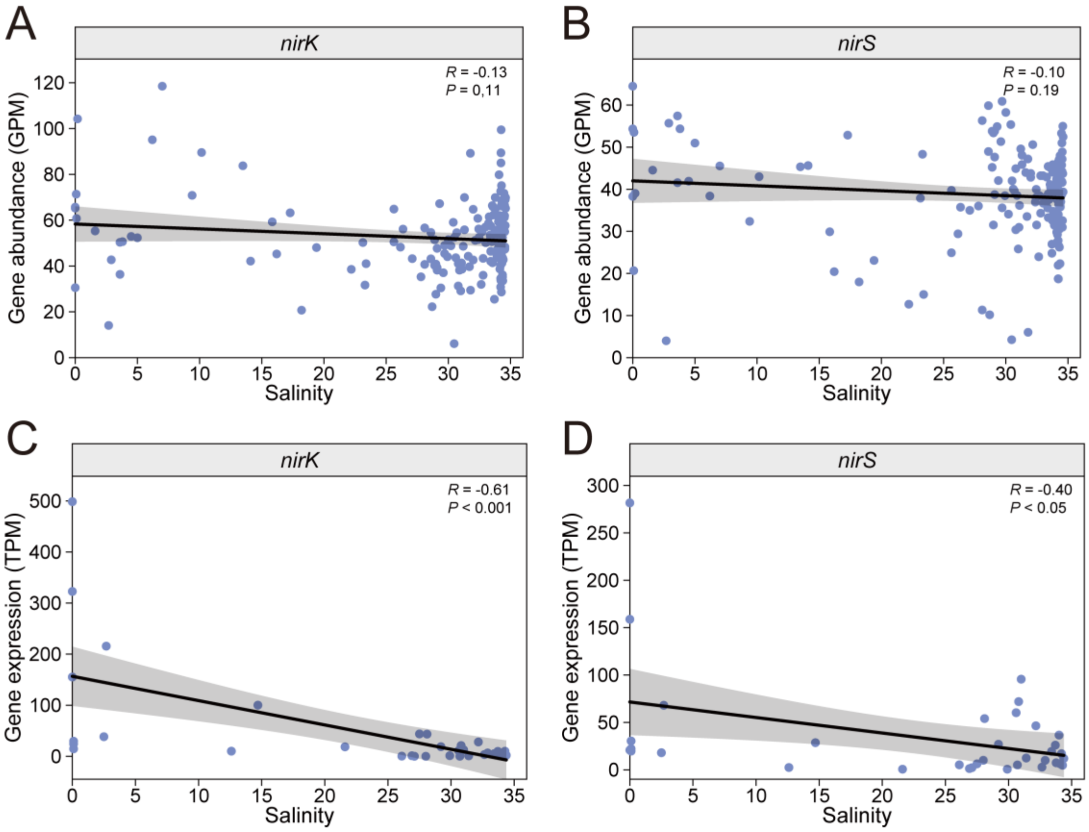

Supplementary Figure 10. Relative abundance and expression of *nirK* and *nirS* genes across salinity gradient. (**A–B**) Relative abundance of *nirK* and *nirS* along the salinity gradient, respectively. (**C–D**) Relative expression of *nirK* and *nirS* along the salinity gradient, respectively. Shaded areas indicate 95% confidence intervals around the fitted regression lines.

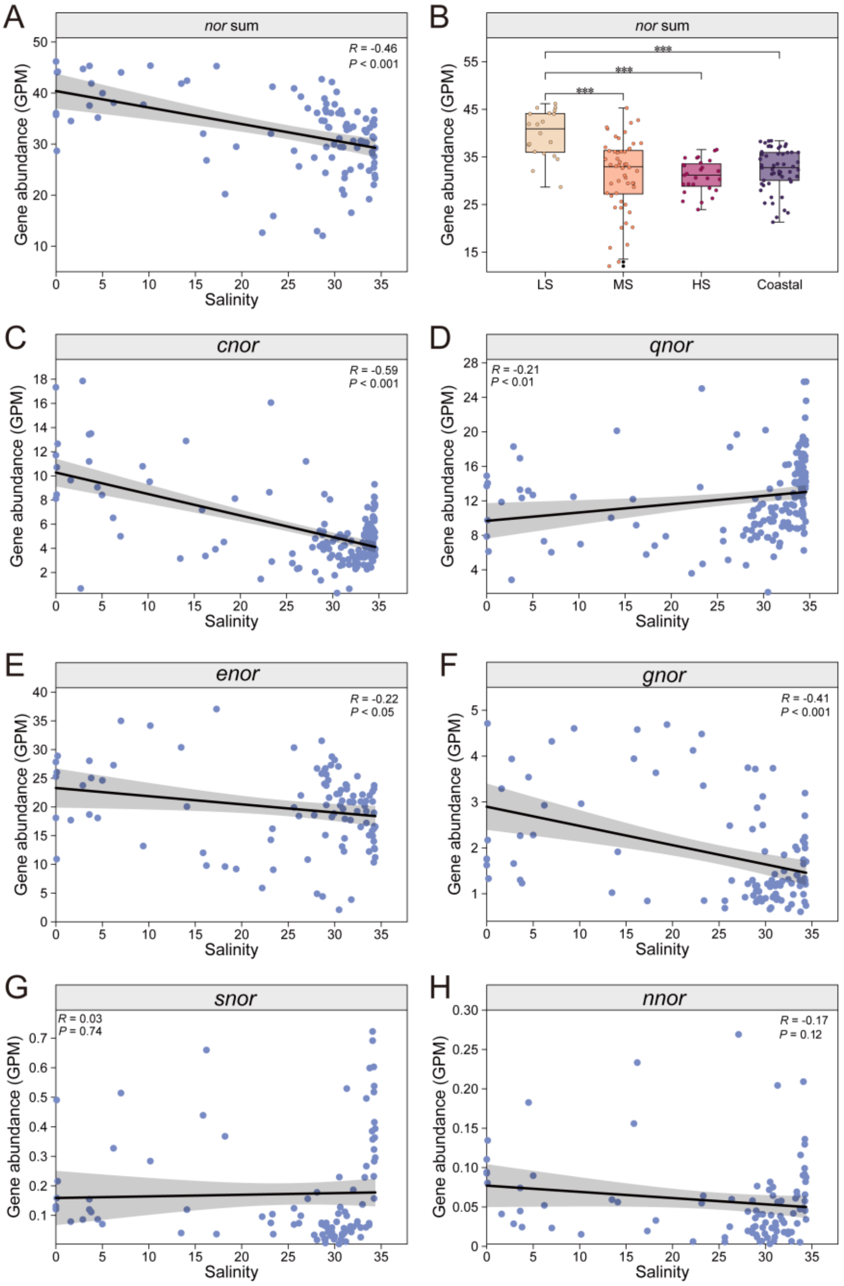

Supplementary Figure 11. Relative abundance of *nor* genes across salinity gradient. (**A**) The summed relative abundance of the six *nor* clades along the salinity gradient. (**B**) Differences in summed *nor* abundance among salinity zones. Relative abundance of (**C**) *cnor*, (**D**) *qnor*, (**E**) *enor*, (**F**) *gnor*, (**G**) *snor*, and (**H**) *nnor* along the salinity gradient. Shaded areas indicate 95% confidence intervals around the fitted regression lines. Statistical significance: * $P < 0.05$; ** $P < 0.01$; *** $P < 0.001$.

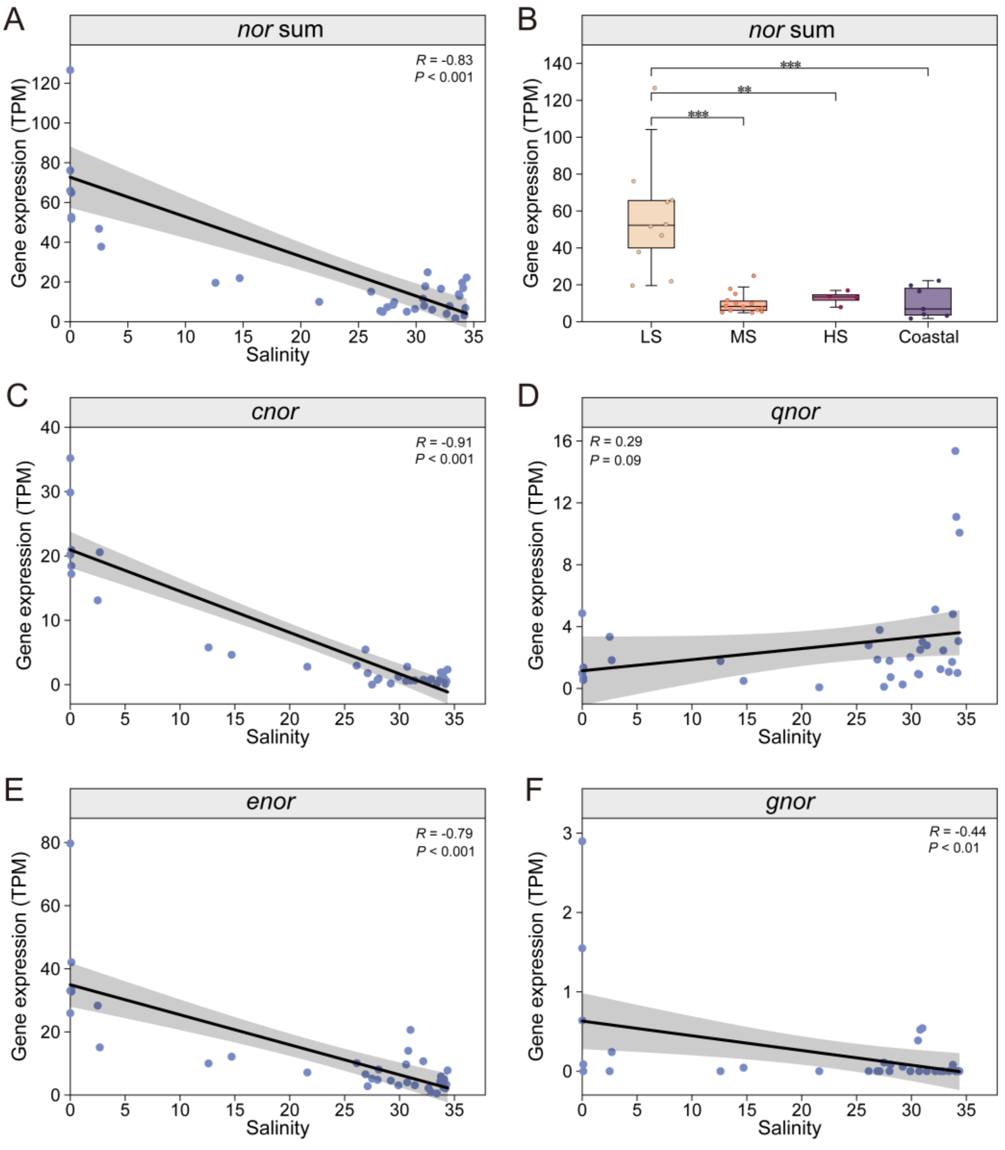

Supplementary Figure 12. Relative expression of *nor* genes across salinity gradient. (**A**) The summed relative expression of the six *nor* clades along the salinity gradient. (**B**) Differences in summed *nor* expression among salinity zones. Relative expression of (**C**) *cnor*, (**D**) *qnor*, (**E**) *enor*, and (**F**) *gnor* along the salinity gradient. Shaded areas indicate 95% confidence intervals around the fitted regression lines. Statistical significance: * $P < 0.05$; ** $P < 0.01$; *** $P < 0.001$.

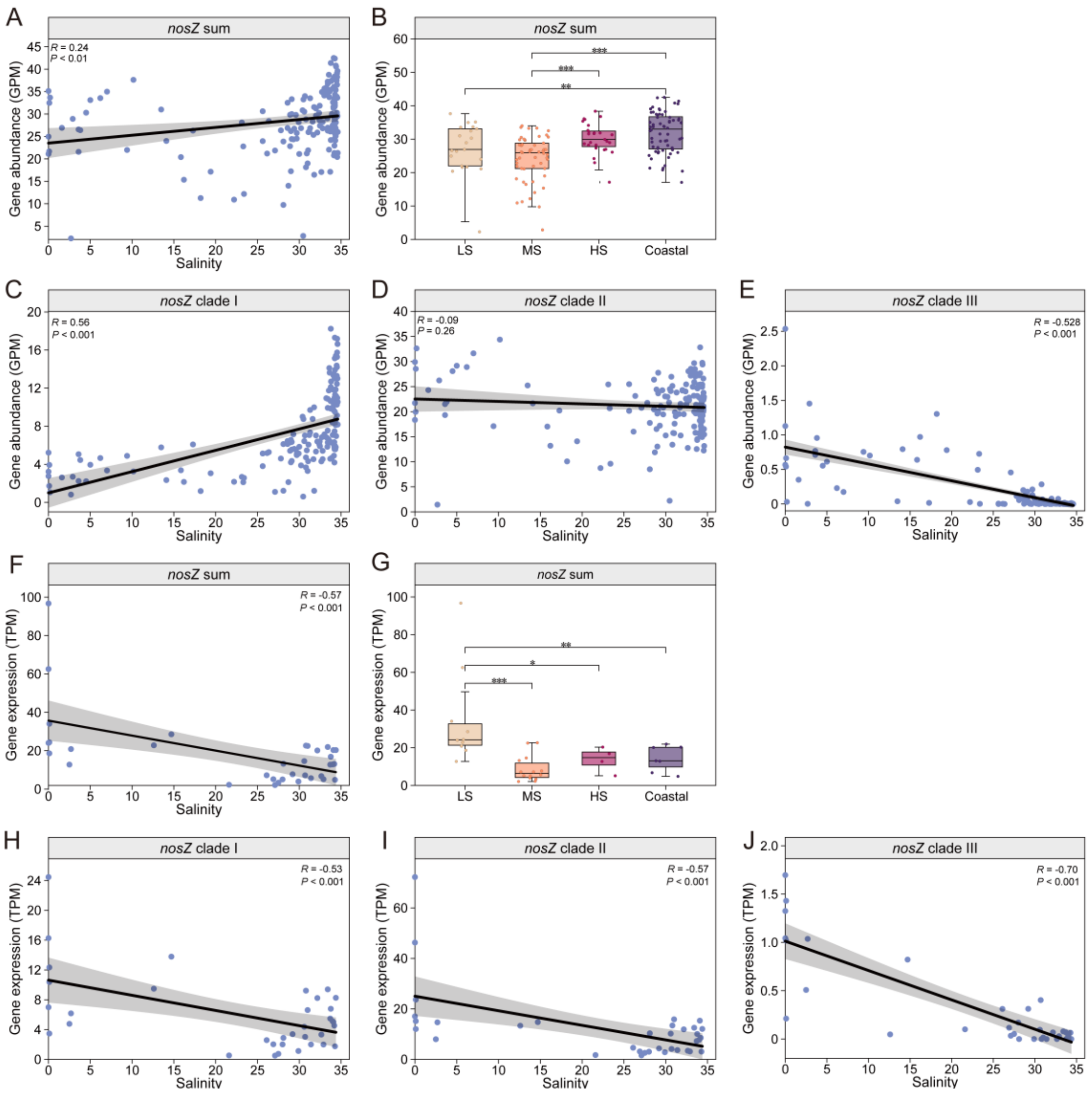

Supplementary Figure 13. Relative abundance and expression of *nosZ* genes across salinity gradient. (**A**) The summed relative abundance of the 3 *nosZ* clades along the salinity gradient. (**B**) Differences in summed *nosZ* abundance among salinity zones. Relative abundance of (**C**) *nosZ* clade I, (**D**) clade II, and (**E**) clade III along the salinity gradient. (**F**) The summed relative expression of the 3 *nosZ* clades along the salinity gradient. (**G**) Differences in summed *nosZ* expression among salinity zones. Relative expression of (**H**) *nosZ* clade I, (**I**) clade II, and (**J**) clade III along the salinity gradient. Shaded areas indicate 95% confidence intervals around the fitted regression lines. Statistical significance: * $P < 0.05$; ** $P < 0.01$; *** $P < 0.001$.

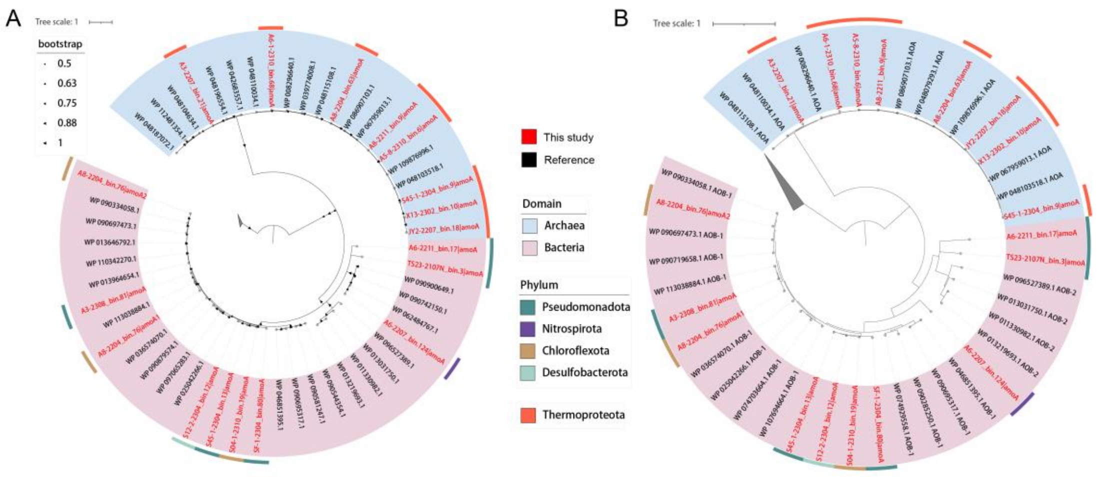

Supplementary Figure 14. Identification of *amoA* genes from the 2,620 MAGs. (**A**) Maximum-likelihood phylogenetic tree of *amoA* genes. Bootstrap values >50% are shown. Scale bar indicates the mean number of substitutions per site. (**B**) Structural tree of *amoA* genes. Branch lengths represent structural distances.

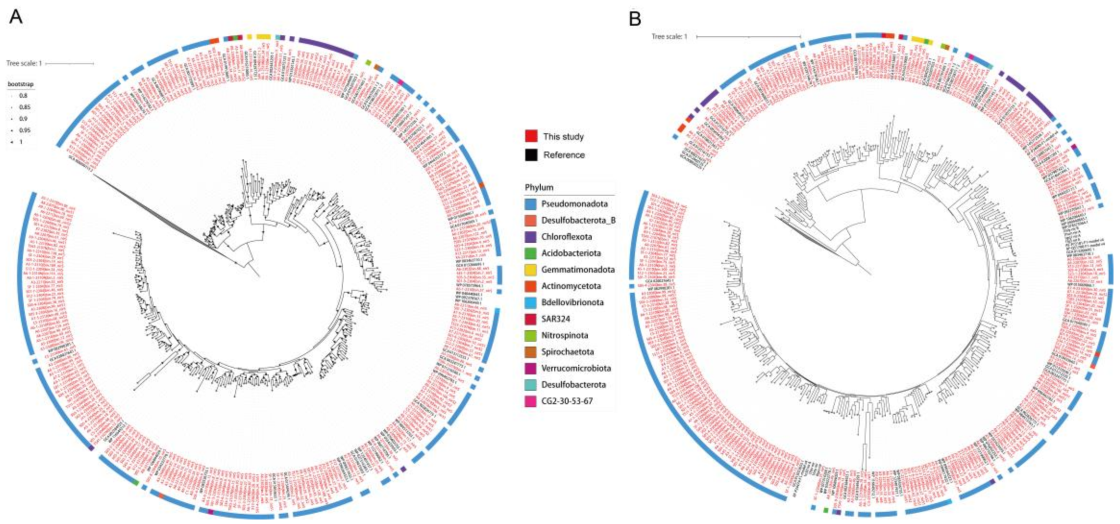

Supplementary Figure 15. Identification of *nirS* genes from the 2,620 MAGs. (**A**) Maximum-likelihood phylogenetic tree of *nirS* genes. Bootstrap values >80% are shown. Scale bar indicates the mean number of substitutions per site. (**B**) Structural tree of *nirS* genes. Branch lengths represent structural distances.

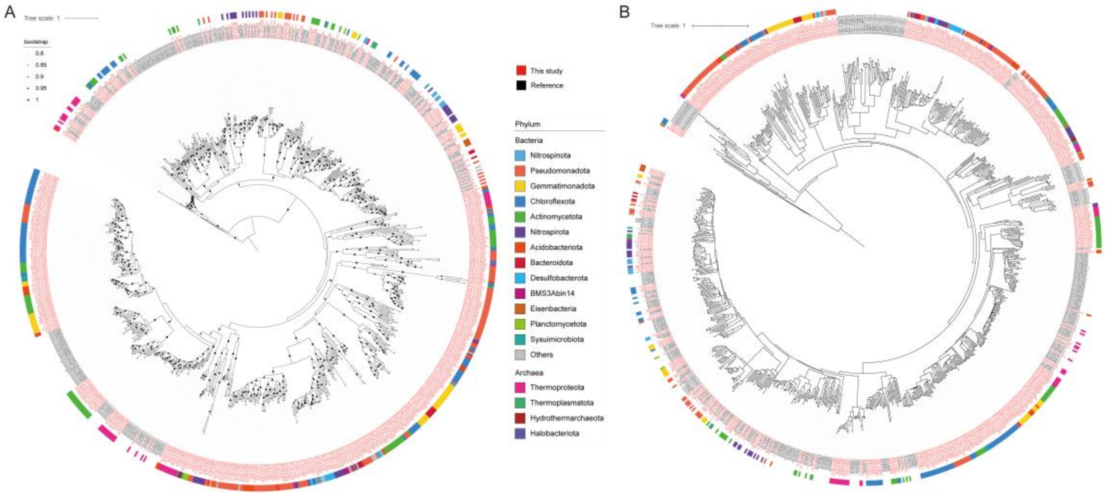

Supplementary Figure 16. Identification of *nirK* genes from the 2,620 MAGs. (**A**) Maximum-likelihood phylogenetic tree of *nirK* genes. Bootstrap values >80% are shown. Scale bar indicates the mean number of substitutions per site. (**B**) Structural tree of *nirK* genes. Branch lengths represent structural distances.

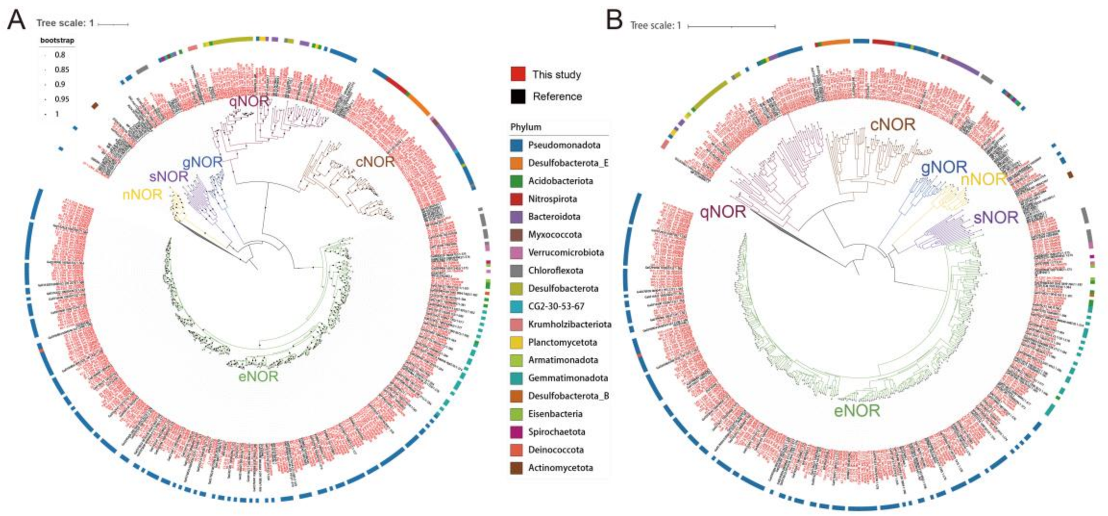

Supplementary Figure 17. Identification of *nor* genes from the 2,620 MAGs. (**A**) Maximum-likelihood phylogenetic tree of *nor* genes. Bootstrap values >80% are shown. Scale bar indicates the mean number of substitutions per site. (**B**) Structural tree of *nor* genes. Branch lengths represent structural distances.

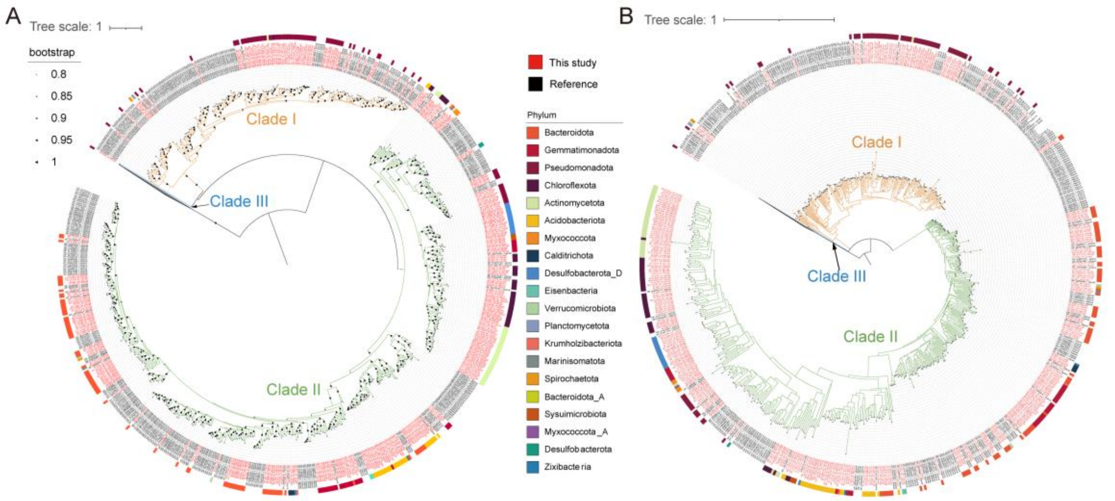

Supplementary Figure 18. Identification of *nosZ* genes from the 2,620 MAGs. (**A**) Maximum-likelihood phylogenetic tree of *nosZ* genes. Bootstrap values >80% are shown. Scale bar indicates the mean number of substitutions per site. (**B**) Structural tree of *nosZ* genes. Branch lengths represent structural distances.

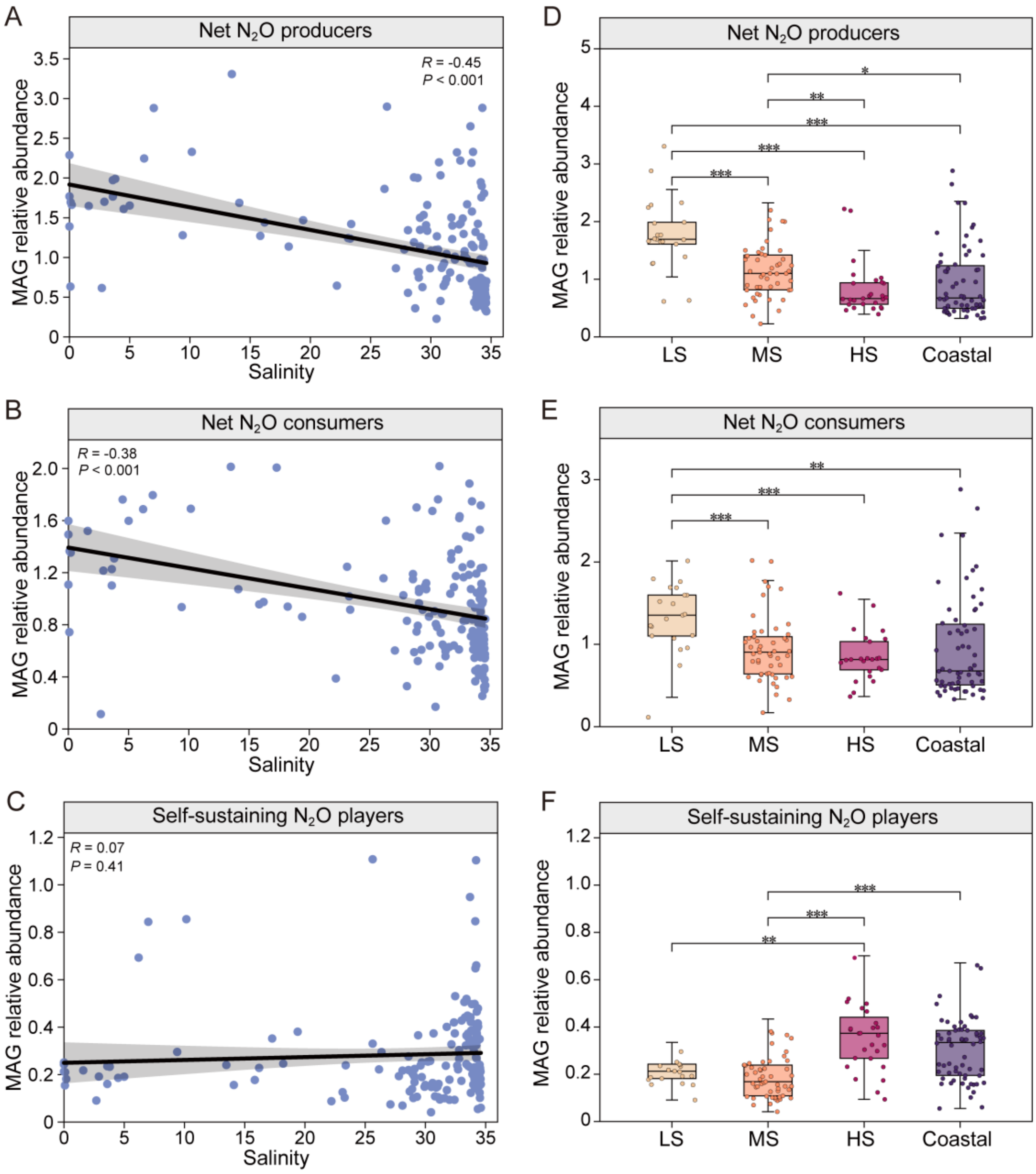

Supplementary Figure 19. Relative abundance of N_2O-related functional groups across salinity gradient. Relative abundance of (**A**) net N_2O producers, (**B**) net N_2O consumers, and (**C**) self-sustaining N_2O players along the salinity gradient. Shaded areas indicate 95% confidence intervals around the fitted regression lines. Differences in the relative abundance of (**D**) net N_2O producers, (**E**) net N_2O consumers, and (**F**) self-sustaining N_2O players among salinity zones. Statistical significance: * $P < 0.05$; ** $P < 0.01$; *** $P < 0.001$.

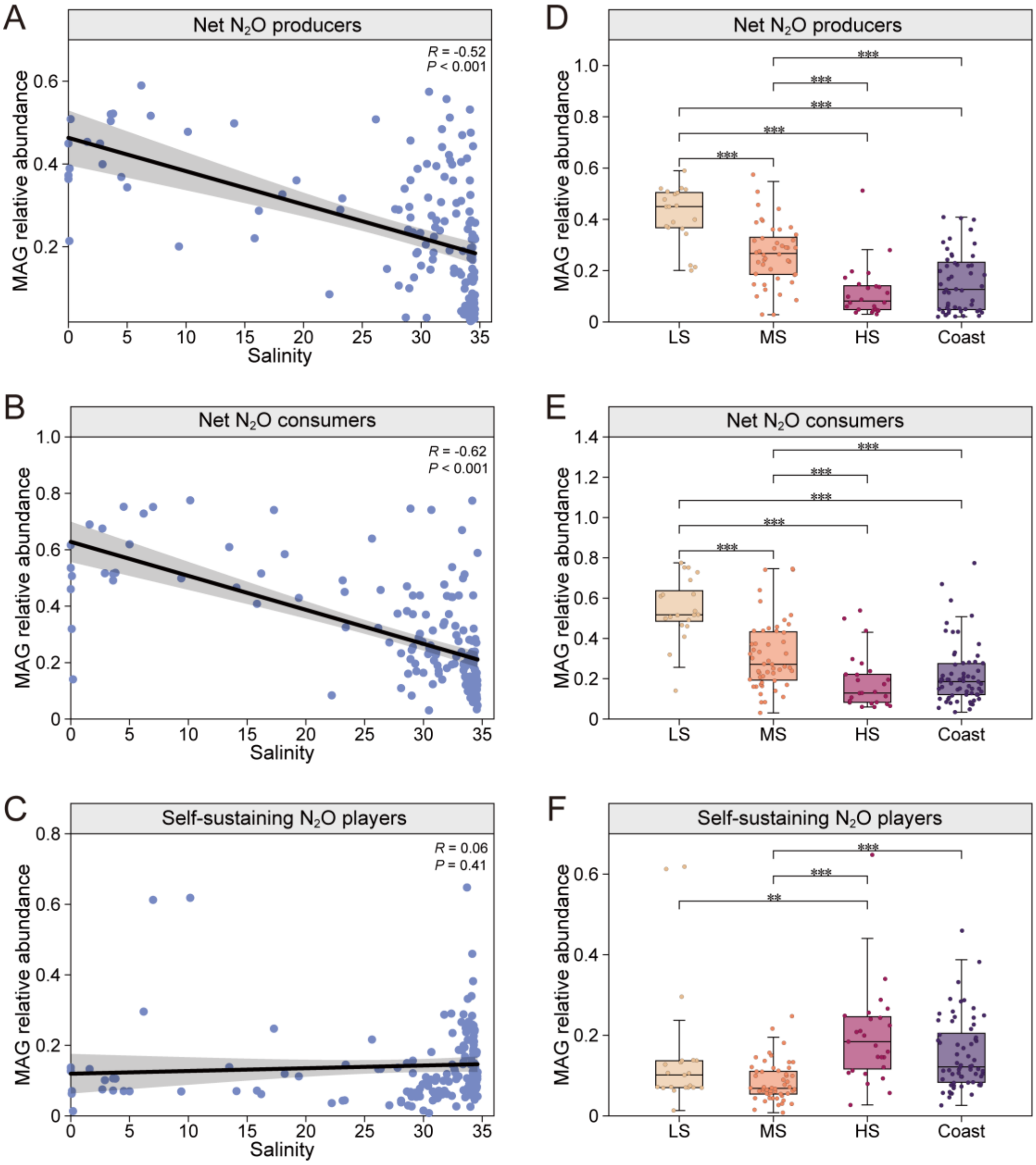

Supplementary Figure 20. Relative abundance of N_2O-related functional groups across salinity gradient based on high-quality MAGs. Relative abundance of (**A**) net N_2O producers, (**B**) net N_2O consumers, and (**C**) self-sustaining N_2O players along the salinity gradient based on the high-quality MAG subset. Shaded areas indicate 95% confidence intervals around the fitted regression lines. Differences in the relative abundance of (**D**) net N_2O producers, (**E**) net N_2O consumers, and (**F**) self-sustaining N_2O players among salinity zones based on the high-quality MAG subset. Statistical significance: * $P < 0.05$; ** $P < 0.01$; *** $P < 0.001$.

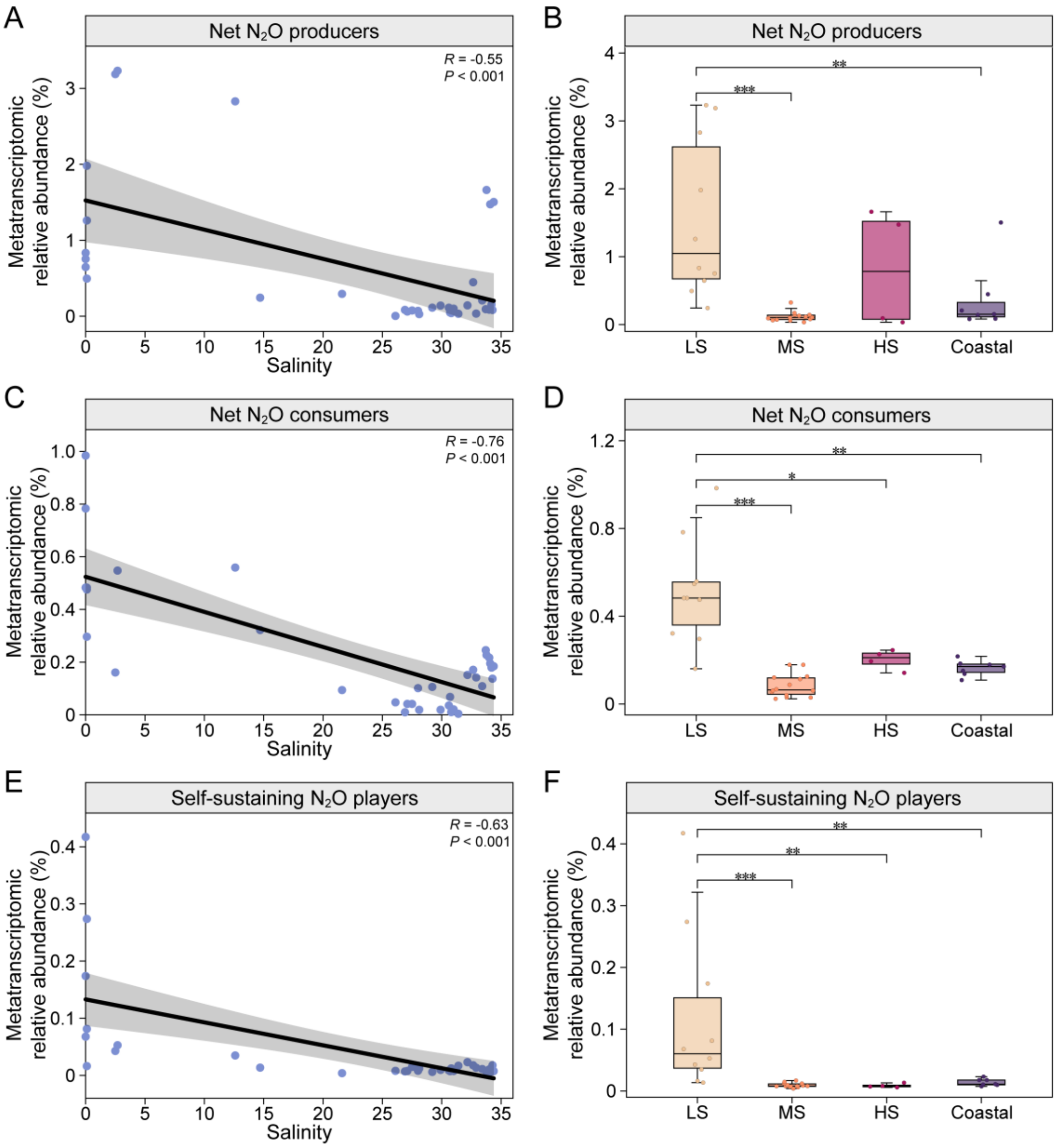

Supplementary Figure 21. Metatranscriptomic relative abundance of N_2O-related functional groups across salinity gradient. Metatranscriptomic relative abundance of (**A**) net N_2O producers, (**B**) net N_2O consumers, and (**C**) self-sustaining N_2O players along the salinity gradient. Shaded areas indicate 95% confidence intervals around the fitted regression lines. Differences in the relative abundance of (**D**) net N_2O producers, (**E**) net N_2O consumers, and (**F**) self-sustaining N_2O players among salinity zones. Statistical significance: * $P < 0.05$; ** $P < 0.01$; *** $P < 0.001$.

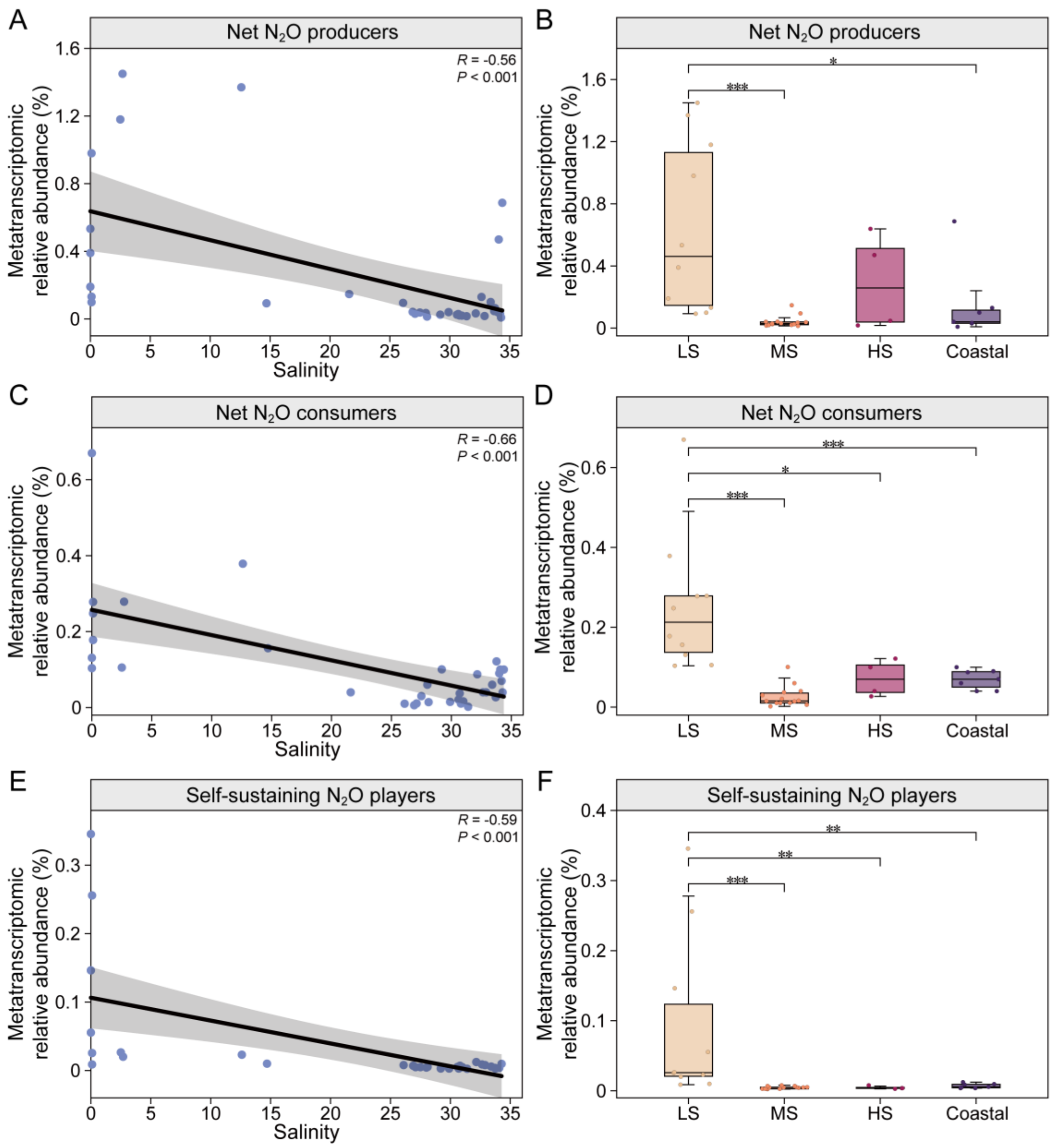

Supplementary Figure 22. Metatranscriptomic relative abundance of N_2O-related functional groups across salinity gradient based on high-quality MAGs. Metatranscriptomic relative abundance of (**A**) net N_2O producers, (**B**) net N_2O consumers, and (**C**) self-sustaining N_2O players along the salinity gradient. Shaded areas indicate 95% confidence intervals around the fitted regression lines. Differences in the relative abundance of (**D**) net N_2O producers, (**E**) net N_2O consumers, and (**F**) self-sustaining N_2O players among salinity zones. Statistical significance: * $P < 0.05$; ** $P < 0.01$; *** $P < 0.001$.

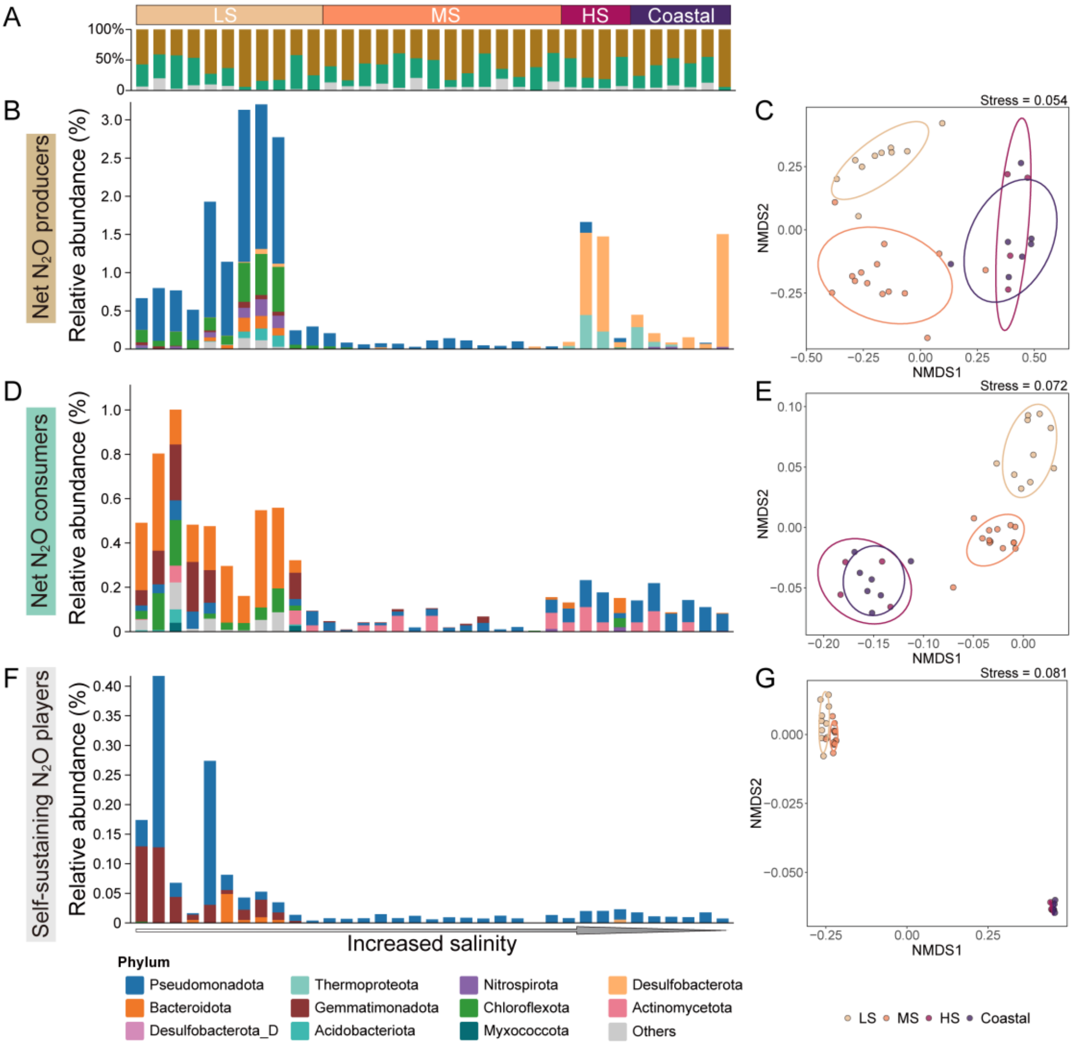

Supplementary Figure 23. Shifts of N_2O-related functional groups across the estuarine-coastal gradient at transcriptional level. (**A**) Relative proportion of the three functional groups within the total N_2O-associated microbial community. (**B**) Community composition and (**C**) non-metric multidimensional scaling (NMDS) analysis of net N_2O producers in 35 sediment samples. (**D**) Community composition and (**E**) NMDS analysis of net N_2O consumers. (**F**) Community composition and (**G**) NMDS analysis of self-sustaining N_2O players.

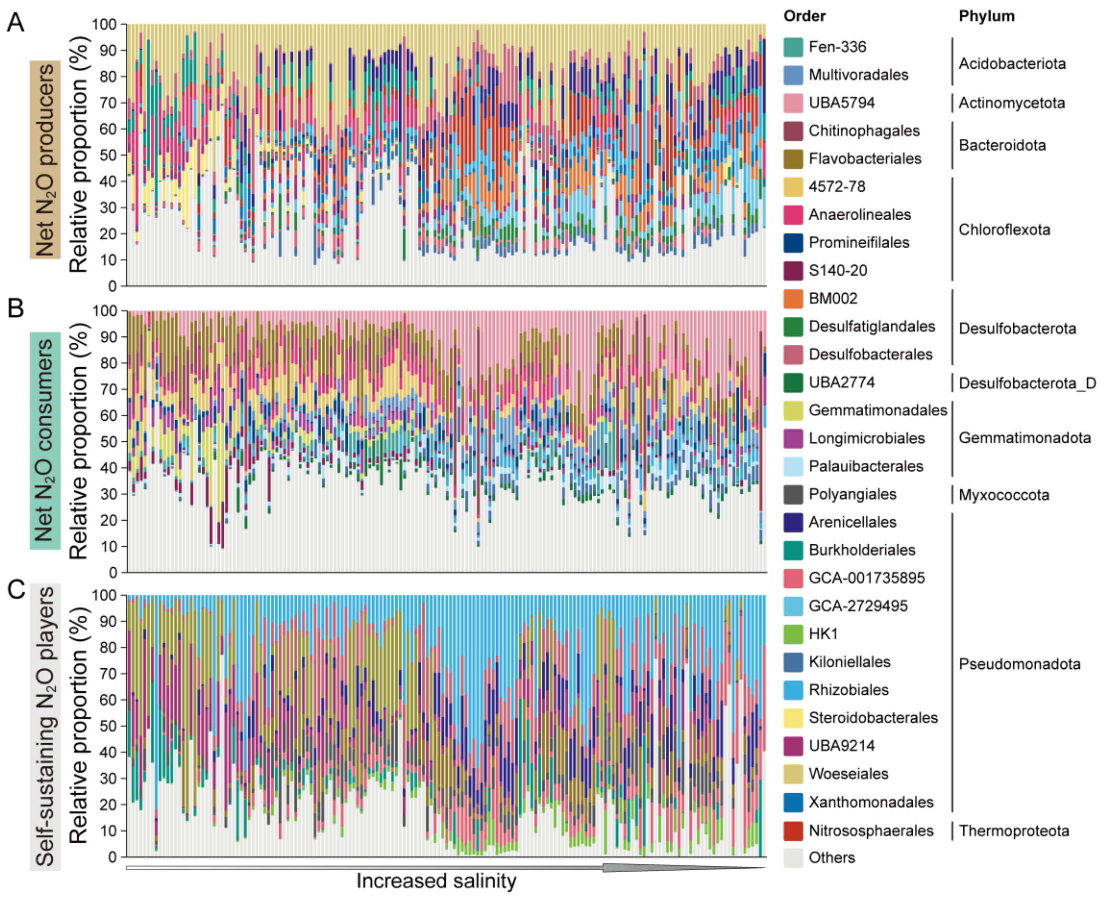

Supplementary Figure 24. Shifts of N_2O-related functional groups at order level across the estuarine-coastal gradient. (**A**) Order-level community composition of net N_2O producers in 165 sediment samples. (**B**) Order-level community composition of net N_2O consumers in 165 sediment samples. (**C**) Order-level community composition of self-sustaining N_2O players in 165 sediment samples.

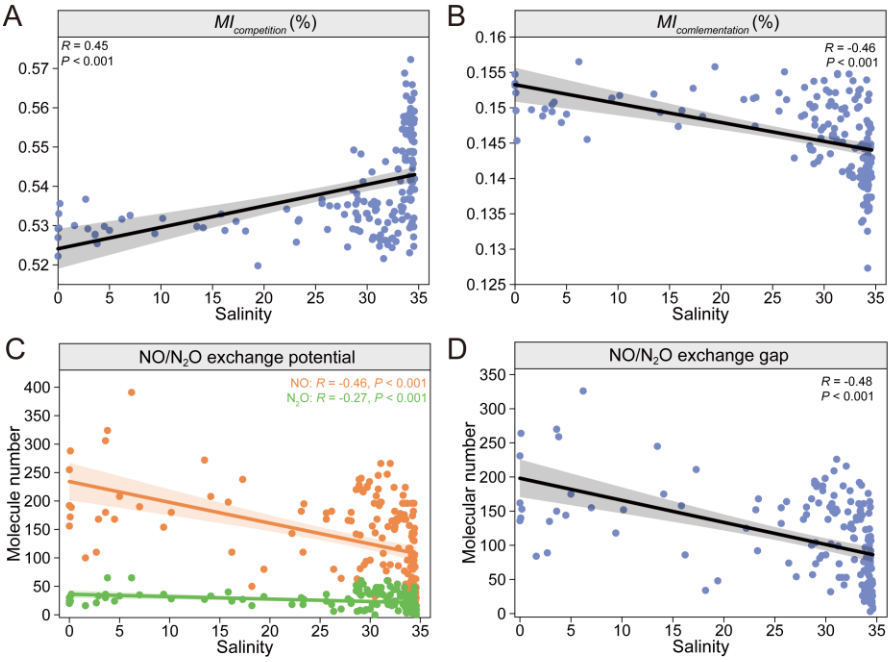

Supplementary Figure 25. Relationships between metabolic interaction indices and salinity. (**A**) Correlation between $MI_{competition}$ and salinity. (**B**) Correlation between $MI_{complementarity}$ and salinity. (**C**) Correlation between NO, N_2O exchange potentials and salinity. (**D**) Correlation between NO/N_2O exchange potential differences and salinity (n = 165). Shaded areas indicate 95% confidence intervals around the fitted regression lines.

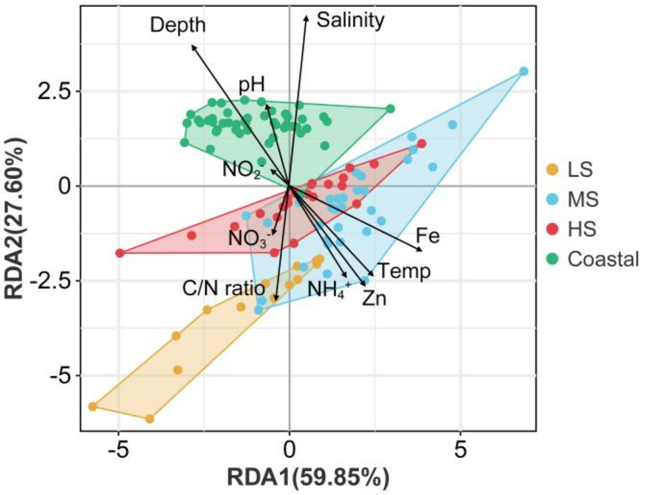

Supplementary Figure 26. Relationship between N_2O-related genes and environmental variables. Redundancy analysis of the relative abundance of N_2O-related genes under environmental variable constraints. The environmental factors shown in the figure are selected through Variance Inflation Factor analysis, retaining only those that are non-collinear.

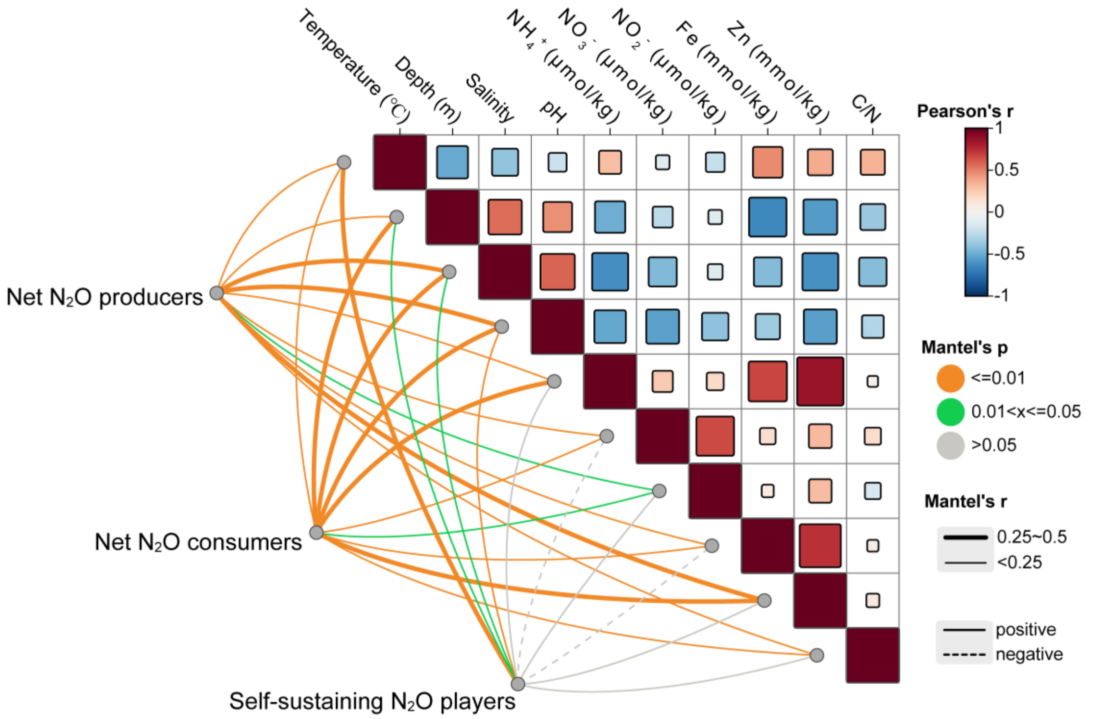

Supplementary Figure 27. Relationships between N_2O-related functional groups and environmental variables. Pairwise correlations among environmental factors are shown using a color gradient representing Pearson correlation coefficients. Mantel tests are used to assess the relationship between N_2O-related functional groups and each environmental factor. Edge width represents Mantel's r statistic, and edge color indicates statistical significance. The environmental factors shown in the figure are selected through Variance Inflation Factor analysis, retaining only those that are non-collinear.

References

1. Gao D et al. Mechanisms responsible for N_2O emissions from intertidal soils of the Yangtze Estuary. *Sci Total Environ* 2020;**716**:137073.
2. Deng D et al. The contribution of anammox to nitrogen removal is greater in bulk soils than in rhizosphere soils in riparian wetlands along the Yangtze River. *Global Biogeochem Cycles* 2023;**37**:e2022GB007576.
3. Chen Q et al. Correspondence between DOM molecules and microbial community in a subtropical coastal estuary on a spatiotemporal scale. *Environ Int* 2021;**154**:106558.
4. Hu Y et al. The imbalance between N_2O production and reduction by multi-microbial communities determines sedimentary N_2O emission potential in the Pearl River Estuary. *Mar Environ Res* 2023;**190**:106119.
